# clusIBD: Robust Detection of Identity-by-descent Segments Using Unphased Genetic Data from Poor-quality Samples

**DOI:** 10.1093/gpbjnl/qzaf055

**Published:** 2025-06-20

**Authors:** Ran Li, Yu Zang, Zhentang Liu, Jingyi Yang, Nana Wang, Jiajun Liu, Enlin Wu, Riga Wu, Hongyu Sun

**Affiliations:** Faculty of Forensic Medicine, Zhongshan School of Medicine, Sun Yat-sen University, Guangzhou 510080, China; School of Medicine, Jiaying University, Meizhou 514015, China; Guangdong Province Translational Forensic Medicine Engineering Technology Research Center, Sun Yat-sen University, Guangzhou 510080, China; Faculty of Forensic Medicine, Zhongshan School of Medicine, Sun Yat-sen University, Guangzhou 510080, China; Guangdong Province Translational Forensic Medicine Engineering Technology Research Center, Sun Yat-sen University, Guangzhou 510080, China; Faculty of Forensic Medicine, Zhongshan School of Medicine, Sun Yat-sen University, Guangzhou 510080, China; Guangdong Province Translational Forensic Medicine Engineering Technology Research Center, Sun Yat-sen University, Guangzhou 510080, China; Faculty of Forensic Medicine, Zhongshan School of Medicine, Sun Yat-sen University, Guangzhou 510080, China; Guangdong Province Translational Forensic Medicine Engineering Technology Research Center, Sun Yat-sen University, Guangzhou 510080, China; Faculty of Forensic Medicine, Zhongshan School of Medicine, Sun Yat-sen University, Guangzhou 510080, China; Guangdong Province Translational Forensic Medicine Engineering Technology Research Center, Sun Yat-sen University, Guangzhou 510080, China; Faculty of Forensic Medicine, Zhongshan School of Medicine, Sun Yat-sen University, Guangzhou 510080, China; Guangdong Province Translational Forensic Medicine Engineering Technology Research Center, Sun Yat-sen University, Guangzhou 510080, China; Faculty of Forensic Medicine, Zhongshan School of Medicine, Sun Yat-sen University, Guangzhou 510080, China; Guangdong Province Translational Forensic Medicine Engineering Technology Research Center, Sun Yat-sen University, Guangzhou 510080, China; Faculty of Forensic Medicine, Zhongshan School of Medicine, Sun Yat-sen University, Guangzhou 510080, China; Guangdong Province Translational Forensic Medicine Engineering Technology Research Center, Sun Yat-sen University, Guangzhou 510080, China; Faculty of Forensic Medicine, Zhongshan School of Medicine, Sun Yat-sen University, Guangzhou 510080, China; Guangdong Province Translational Forensic Medicine Engineering Technology Research Center, Sun Yat-sen University, Guangzhou 510080, China

**Keywords:** Identity-by-descent, Kinship inference, Unphased genetic data, Poor-quality sample, Algorithm

## Abstract

The detection of identity-by-descent (IBD) segments is widely used to infer relatedness in many fields, including forensics and ancient DNA analysis. However, existing methods are often ineffective for poor-quality DNA samples. Here, we propose a method, clusIBD, which can robustly detect IBD segments using unphased genetic data with a high rate of genotyping error. We evaluated and compared the performance of clusIBD with that of IBIS, TRUFFLE, and IBDseq using simulated data, artificial poor-quality materials, and ancient DNA samples. The results show that clusIBD outperforms these existing tools and could be used for kinship inference in fields such as ancient DNA analysis and criminal investigation. clusIBD is publicly available at GitHub (https://github.com/Ryan620/clusIBD/) and BioCode (https://ngdc.cncb.ac.cn/biocode/tool/BT007882).

## Graphical abstract

**Figure:**
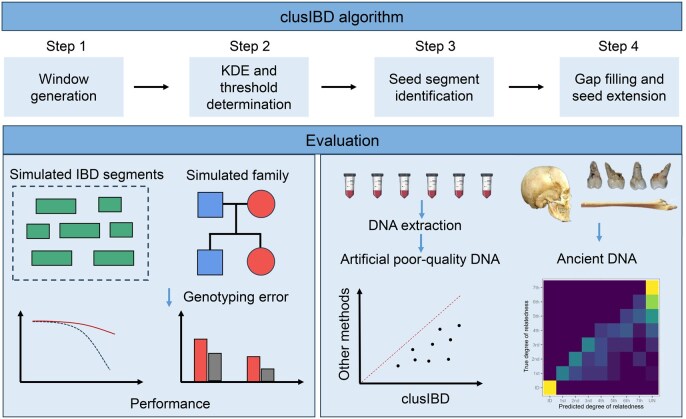

## Introduction

Inferring relatedness from genomic data is an essential component in many fields, such as genetic association studies, population genetics, forensics, archaeology, and ecology [1–6]. For example, such inferences help avoid spurious signals in genetic association studies, as unknown kinship among the cases or controls may inflate the false-positive rate [4]. In population genetic analyses, kinship needs to be taken into account, and in some instances relatives should be removed from the sample before other analyses are performed, such as those investigating population structure, human evolutionary history, times of population splitting, migration rates, and mating patterns among individuals [7,8]. In forensic genetics, familial searching and investigative genetic genealogy can help find the perpetrators of crimes and identify unknown deceased individuals through the DNA of their relatives [9–11], which extends the investigative value of current forensic DNA databases and direct-to-consumer (DTC) databases [12]. Kinship inference also has important applications in the field of archeology. For example, analysis of ancient DNA can provide insights into the demographic structure, as well as the culture and social hierarchy, of individuals from ancient times [5,13,14]. Meanwhile, for non-humans, studying patterns of kinship relationships among individuals in a local population is also useful for characterizing the social organization and population biology [6].

Many methods have been proposed for kinship inference, which mainly involve estimating the proportion of the genome shared identity-by-descent (IBD) between individuals [1,15,16]. Among these methods, allele frequency-based approaches [17,18] are computationally efficient and have been widely used to detect cryptic relatedness in large genomic datasets [19–21]. However, these methods can only accurately infer close relationships (generally up to the third degree) [18,22] and are less accurate at identifying distant relatives (*e.g.*, sixth- and seventh-degree relatives) [23]. Furthermore, allele frequency-based approaches may be biased in admixed samples due to admixture linkage disequilibrium [24]. In contrast, IBD segment-based methods are generally more accurate and can identify more distant relationships than methods based on the allele frequencies of independent markers [23]. The key idea behind IBD segment detection is haplotype frequency. Specifically, if the frequency of a shared haplotype (in phased genotype data) or a half-shared segment (in unphased genotype data) is so low that it is unlikely to be observed twice in independently sampled individuals, the presence of an IBD segment can be inferred [25].

Haplotype-based methods are generally more accurate at detecting short IBD segments than genotype-based methods [26]. However, haplotype-based methods can break up a long IBD segment into a series of shorter ones when phasing errors are present in the data [27], which can be further exacerbated by genotyping errors. For instance, Turner et al. [28] demonstrated that hap-ibd, a well-known haplotype-based method, performed worse than frequency- and genotype-based methods (KING [18] and IBIS [29]) when using genotype data with error rates exceeding 1%. Furthermore, phasing often requires a genetic map and reference panels, which may not be available for the dataset of interest. In contrast, genotype-based methods do not require phasing, and can tolerate a higher level of genotyping error [28].

Several excellent tools have been developed to detect IBD segments using unphased genotype data. These include IBIS [29], IBDseq [30], and TRUFFLE [31], which are fast and accurate when applied to DNA samples of relatively high quality and quantity. However, these tools may be inadequate for challenging materials, such as ancient human remains, crime scene samples, and tissues collected noninvasively from wild animals (*e.g.*, feces, hair, and urine) [28,32,33]. Only a small quantity of DNA is typically isolated from such samples, and it is also often mixed with nonhost DNA. For example, only around 1% of the DNA extracted from fecal samples is endogenous to the donor animal [34], and ancient DNA libraries often contain less than 1% endogenous DNA, with the majority of sequencing capacity taken up by environmental DNA [35]. As such, the generation of genetic data from these low-quality DNA samples is particularly susceptible to error. Ozga et al. [36] showed that allelic dropout rates ranged from 0.66% to 18.42% in genome-scale data generated through shotgun sequencing, whole-genome capture sequencing, and whole-exome capture sequencing using feces, urine, dentin, and dental calculus from wild eastern chimpanzees. It has also been reported that when the amount of input DNA is less than 20 pg, the rate of genotyping error can even exceed 20% when using single nucleotide polymorphism (SNP) microarrays [37]. When the genotyping error is low (< 1%), existing IBD segment detection methods outperform allele frequency-based approaches (*e.g*., KING) for inferring close and distant relationships, but become less robust as genotyping error increases (1%–5%) [28].

To overcome these obstacles, we here propose clusIBD, a method that detects IBD segments by identifying clusters of regions featuring the opposite homozygote or mismatch at low rates between samples using unphased genetic data. We evaluated and compared the performance of clusIBD with that of IBIS, TRUFFLE and IBDseq using simulated families, artificial challenging materials, and a five-generation family from chambered tombs dating back to Early Neolithic Britain [13]. The results demonstrate that clusIBD can robustly detect IBD segments from materials of varying quality and quantity and may be a promising tool in forensic genetics and ancient DNA analysis.

## Method

### clusIBD algorithm

**Figure 1:**
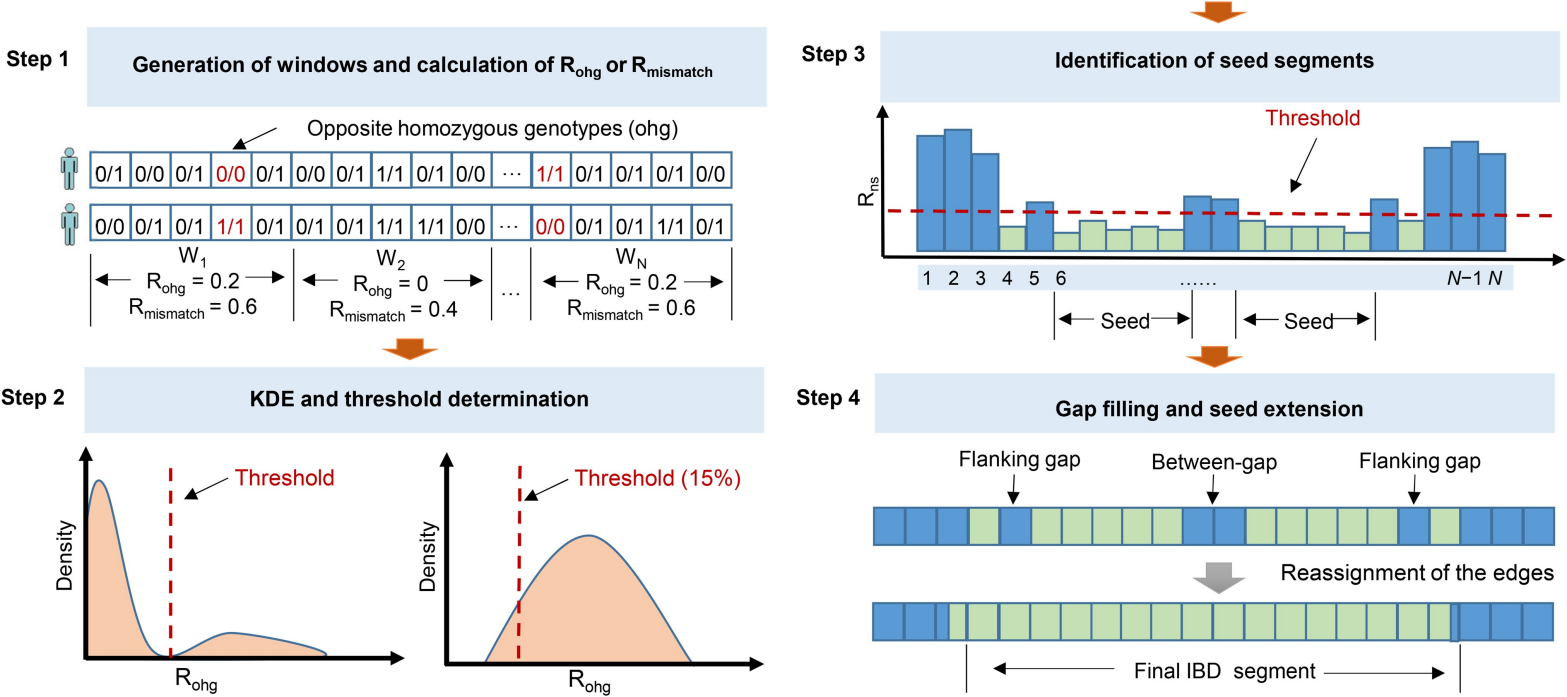
Illustration of clusIBD algorithm Step 1: Obtain non-overlapping windows and calculate the rates of opposite homozygous genotypes (R_ohg_) for IBD1 or mismatch rates (R_mismatch_) for IBD2. Step 2: Perform KDE and determine a threshold that can be used to distinguish windows from either IBD or non-IBD segments. Step 3: Identify consecutive windows, all of which are below the threshold, and regard them as a seed segment. Step 4: Fill the gap between two seed segments and extend it on both sides if the flanking gaps are smaller than a predefined value. Finally, reassign the start and end positions and report the IBD segments. IBD, identity-by-descent; IBD1, IBD in one copy of the genome; IBD2, IBD in both copies of the genome; KDE, kernel density estimation.

The clusIBD algorithm is illustrated in **Figure 1**. It works on unphased, bi-allelic SNPs with a minor allele frequency (MAF) above 0.05. All the markers are arranged by physical position, and are divided into non-overlapping windows with a fixed number of SNPs. First, we consider regions where two individuals share only one haplotype (IBD1). We then calculate the rate of opposite homozygous genotypes (R_ohg_), within each window, and obtain a large number of R_ohg_ values, typically more than a thousand, for each pair of individuals. For unrelated individuals, the R_ohg_ values depend on the MAFs of the SNP markers used and can be estimated as $2p^{2}q^{2}$ for each marker [18], where p and q are the frequencies of the reference and alternative alleles, respectively. In the absence of genotyping errors and mutations, windows from IBD segments will all have R_ohg_ values of zero because they share at least one allele at each site. However, when genotyping errors are introduced, the R_ohg_ values of IBD segments are no longer all zero, but are expected to be smaller than those of non-IBD segments.

By exploiting this difference, a threshold (t) of R_ohg_ is determined and used to distinguish windows from either IBD or non-IBD segments. We first estimate the distribution of these R_ohg_ values using kernel density estimation (KDE). If the two individuals have both IBD1 and non-IBD segments, we will see two peaks: the smaller one (near the y-axis) corresponding to windows of IBD segments (IBD peak) and the larger one corresponding to non-IBD segments (non-IBD peak; Figure 1). We will also see a valley between the two peaks, which is an ideal threshold (t_valley_) for distinguishing windows from either IBD or non-IBD segments. Then, t_valley_ can be determined using the R_ohg_ value corresponding to the inflection point (*i.e.*, the point at which the probability density starts to increase after decreasing). However, if the two individuals have unbalanced amounts of IBD and non-IBD segments, the IBD peak may be so small that it gets buried in the non-IBD peak. This is often observed in relationships of the fourth degree or more distant (Figure S1). To address this issue, we set a threshold corresponding to the 15th quantile (t_15th_) of the R_ohg_ values obtained for each pair of individuals with only one peak. Another problem arises when dealing with parent–child relationships, monozygotic twins, and genetic data from the same individual. Since they are homogeneous across the genome, we will also see only one peak, and theoretically only 15% of the genome can be identified. To overcome this problem, we randomly select several samples from the input data before the general analysis and calculate the heterozygote rate (*He*) for each window per sample. Since the expected *He* value is 2pq, a universal threshold (t_universal_) can be estimated as $t_{\mathrm{universal}}=\frac{\;{\mathrm{quantile}(He,\;15\%)}^{2}}{2}$. The final threshold is the highest value among t_valley_, t_15th_, and t_universal_. Because clusIBD can adjust the threshold dynamically based on the genotyping error rate of the input data, it can robustly detect IBD segments from materials containing DNA of varying quality and quantity.

Some windows of non-IBD segments may have R_ohg_ values below the defined threshold. However, the probability of observing a region with a number of consecutive windows all below the threshold is very low. If such a region is identified, it is likely to be a part of an IBD segment and we call it a seed segment [26]. Owing to stochastic effects, the R_ohg_ values of some windows of IBD segments may also exceed the defined threshold, thus resulting in a series of interrupted segments. These gaps are addressed by using a gap-filling and seed-extension strategy. Specifically, if two seed segments are close to each other, we merge them and the gaps between them into one long segment (*i.e.*, gap-filling), considering that the probability of observing two recombination events within a small region is low. Similarly, if a long seed segment and small gaps on either side are identified, we extend the boundary of the seed segment by skipping the gaps (*i.e.*, seed extension). A similar process has also been used in alternative tools [26,32]. This process is very useful for error control and makes clusIBD quite robust, especially when identifying IBD segments using genetic data with high genotyping errors (see Results). Finally, the edges of a segment are reassigned: the last site with opposite homozygous genotypes in the first window is used as the final start position, while the first site with opposite homozygous genotypes in the last window is used as the final end position.

The same procedure can be used, with only some minor modifications, to infer the IBD2 segment. For IBD2 estimation, the rate of mismatches (R_mismatch_) or different genotypes, is calculated instead of R_ohg_. Since the main factor interfering with IBD2 is IBD1 rather than IBD0, we use a different procedure to determine the universal threshold ($t_{\mathrm{universal}}^{\left(2\right)}$). As IBD1 segments share one allele by descent at each site, the probability of having different genotypes is expected to be 2pq (*i.e.*, *He*). Therefore, $t_{\mathrm{universal}}^{\left(2\right)}$ can be estimated as $t_{\mathrm{universal}}^{\left(2\right)}=\mathrm{quantile}(He,\;15\%).$

### Implementation

We have implemented clusIBD in Python, including support for multithreading, and released it as open-source software (https://github.com/Ryan620/clusIBD/). clusIBD accepts the PLINK binary ped format (*i.e.*, bed, bim, and fam files) as input and outputs IBD segments in a human-readable text format. Two search modes are implemented: “within-search” and “across-search”. The former refers to inferring IBD segments for all the pairs within a database (one input file), whereas the latter works by inferring IBD segments between two individuals from different databases (two input files). The latter is often performed when data from one or more individuals of interest are used as queries to search a large database to identify their potential relatives [38].

### Simulated data

Haplotype data from five representative populations of five major super populations were extracted from the 1000 Genomes Project dataset (https://www.internationalgenome.org/). These populations are Utah residents with north and west European ancestry (CEU) representing Europeans, Han Chinese in Beijing (CHB) representing East Asians, Gujarati Indians from Houston (GIH) representing South Asians, those of Mexican ancestry from Los Angeles (MXL) representing admixed Americans, and Yoruba in Ibadan (YRI) representing Africans. For each population, we only included bi-allelic autosomal SNPs with an MAF exceeding 0.05 and with a physical distance greater than 2 kb. We then randomly selected a subset of these SNPs to create a reference panel of approximately 400,000 SNPs. Based on these panels, artificial IBD segments and family data were simulated. Specifically, we randomly selected a pair of individuals and a region of varying size, and then a segment from one individual was injected into the same positions of another individual, thus generating an artificial IBD segment (IBD1). The phase state was discarded, and four levels of genotyping errors were introduced: 0%, 1%, 5%, and 10%. Pedigrees were simulated using Ped-sim [39] with sex-average genetic map [40]. From the simulated pedigree, different relationships can be obtained, including parent–child, full siblings, and second-to-seventh-degree relatives, as well as unrelated pairs.

### Artificial poor-quality DNA

To test the performance of clusIBD on challenging materials, we generated a series of low-quantity and low-quality samples. Specifically, whole-blood samples were collected with informed consent from six donors from a multi-generation family (Figure S2). The DNA was then extracted using a QIAamp DNA Blood Mini Kit (Catalog No. 51106, Qiagen, Hilden, Germany) and quantified on a Qubit 3.0 fluorometer (Thermo Fisher Scientific, South San Francisco, CA) according to the manufacturer’s instructions. We further diluted these samples by adding nuclease-free water to mimic low-quantity DNA, and the final amounts of DNA were 100 ng, 10 ng, 1 ng, 0.5 ng, and 0.1 ng for each sample. We also isolated 100 ng of DNA and degraded it using a Covaris M220 focused ultrasonicator (Covaris, Woburn, MA). By controlling the duty factor and treatment time, DNA with four levels of degradation was obtained, with average fragment sizes of approximately 2500 bp, 800 bp, 400 bp, and 150 bp, respectively. These samples were then genotyped using the Infinium Asian Screening Array (ASA; Illumina, San Diego, CA). SNPs with an MAF of less than 0.05, or located on a sex chromosome were excluded, thus resulting in approximately 300,000 SNPs.

### Ancient DNA

Thirty-five individuals who lived approximately 5700 years ago and were entombed at the Hazleton North long cairn were characterized in detail by Fowler et al. by combining archeological and genetic data [13]. Pedigree reconstruction indicated that these samples consisted of 27 individuals from a 5-generation lineage and 8 unrelated individuals. We downloaded the aligned sequencing data (bam files) from the European Nucleotide Archive (ENA: PRJEB46958). SNP calling was performed at 1,233,013 sites of the 1240K panel using SAMtools [41]. We also downloaded genetic data of present-day individuals from England from the 1000 Genome Project dataset. We then removed SNPs with more than 50% missing calls and MAFs of less than 0.05, and individuals with more than 50% missing sites. After these filtering steps, the final set consisted of 30 individuals genotyped at 361,399 SNPs.

### Relatedness classification

For each pair of individuals, we calculated the kinship coefficient (θ) [42], which denotes the probability that a randomly selected allele in one individual is IBD with a randomly selected allele from the same genomic position of another individual. θ can be calculated as $\theta =\frac{k2}{2}+\frac{k1}{4}$, where k2 and k1 denote the proportions of the genomes that two individuals share as IBD2, and IBD1, respectively. Thus, with reported IBD segments, θ can also be simply estimated as $\theta =\frac{L(\mathrm{IBD}2)}{2\times L(\mathrm{genome})}+\frac{L(\mathrm{IBD}1)}{4\times L(\mathrm{genome})}$, where L(IBD1), L(IBD2), and L(genome) are the total lengths of IBD1 segments, IBD2 segments, and the whole genome, respectively. The expected values of θ for different relationships are listed in **Table 1**. Following a decision-making process similar to KING [18], we inferred the degree of relatedness for each pair of individuals using the criteria in Table 1, and pairs with $\theta <\frac{1}{2^{17/2}}$ were assigned as unrelated.

**Table 1:** The expected values of kinship coefficients and inference criteria for different relationships

Degree of relatedness	Expected θ	Cutoff
Monozygotic twin (identical sample)	1/2	$\geq \frac{1}{2^{3/2}}$
First degree (1st)	1/4	$\left(\frac{1}{2^{5/2}},\frac{1}{2^{3/2}}\right)$
Second degree (2nd)	1/8	$\left(\frac{1}{2^{7/2}},\frac{1}{2^{5/2}}\right)$
Third degree (3rd)	1/16	$\left(\frac{1}{2^{9/2}},\frac{1}{2^{7/2}}\right)$
Fourth degree (4th)	1/32	$\left(\frac{1}{2^{11/2}},\frac{1}{2^{9/2}}\right)$
Fifth degree (5th)	1/64	$\left(\frac{1}{2^{13/2}},\frac{1}{2^{11/2}}\right)$
Sixth degree (6th)	1/128	$\left(\frac{1}{2^{15/2}},\frac{1}{2^{13/2}}\right)$
Seventh degree (7th)	1/256	$\left(\frac{1}{2^{17/2}},\frac{1}{2^{15/2}}\right)$
Unrelated	0	$<\frac{1}{2^{17/2}}$

## Results

### IBD segment detection

First, we used simulated IBD segments to evaluate the performance of clusIBD in detecting IBD segments of different lengths (10–30 Mb) and with different levels of genotyping error. It is worth noting that genetic distance is often a better unit to use for length-based IBD detection, but a genetic map may not be available for the dataset of interest (*e.g*., ancient DNA samples). Based on our established reference panels, 500 artificial IBD segments were simulated for each length and error group as described in the Method section. Since we know the true breakpoints of each ground-truth IBD segment, we can compare them with the reported IBD segments to evaluate the performance. However, the comparison is not straightforward because the reported segments may be partially true, or only part of the ground-truth IBD segments may be reported. Therefore, we used the four metrics defined by Tang and colleagues [43], namely, recall and power to evaluate the ground-truth IBD segments, and accuracy and length accuracy (len.accuracy) to evaluate the reported IBD segments. We also compared the performance of clusIBD with the other three methods, *i.e.*, IBIS, TRUFFLE, and IBDseq. The default options for all four methods were used throughout the analyses, unless otherwise specified.

**Figure 2:**
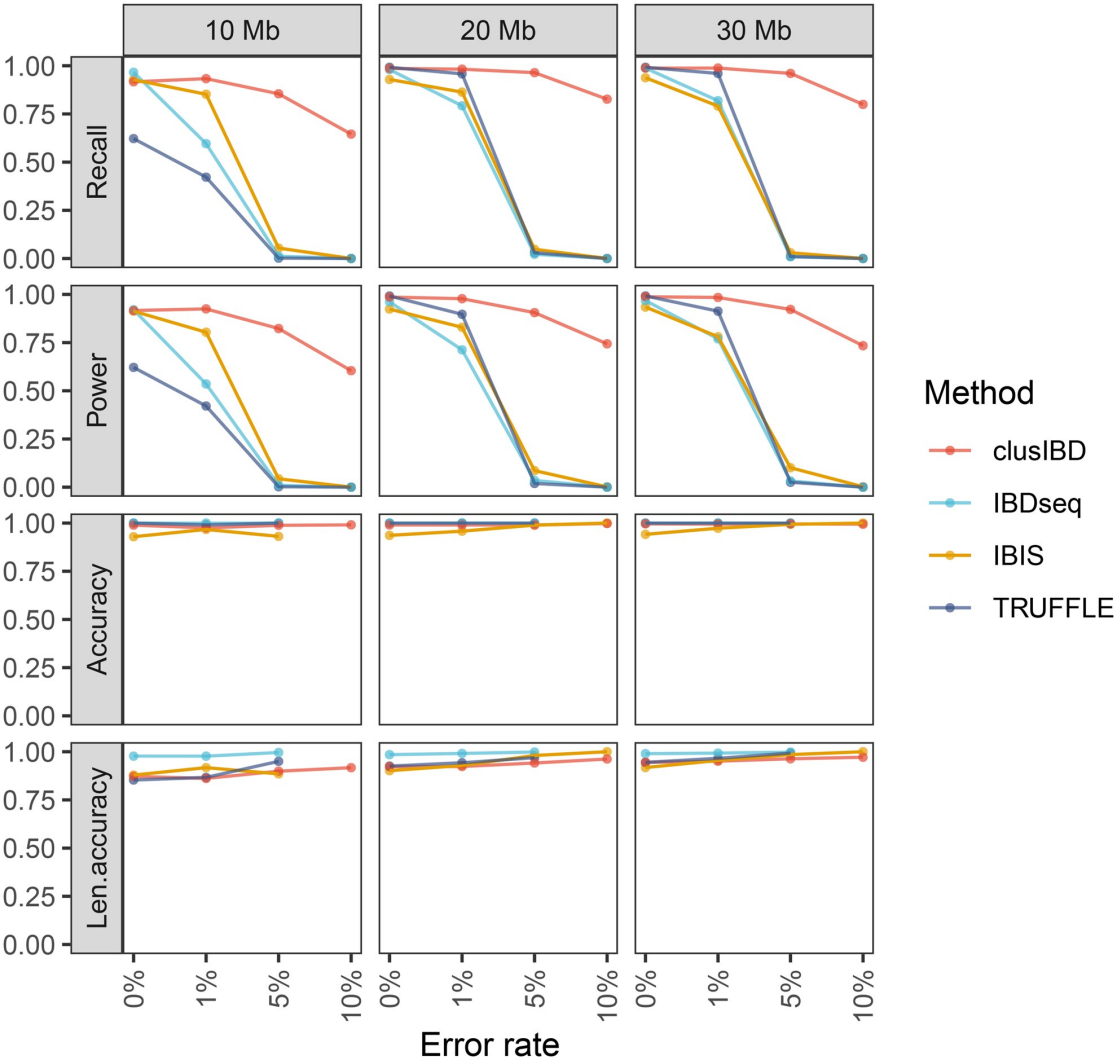
Performance of clusIBD, IBDseq, IBIS, and TRUFFLE for detecting IBD segments of different lengths and with different levels of genotyping error Recall is the proportion of ground-truth IBD segments 50% or more of which could be covered by any one reported IBD segment. Power measures the proportion of lengths of ground-truth IBD segments that are covered by the best-reported IBD segment. Accuracy is the proportion of reported IBD segments 50% or more of which could be covered by any one ground-truth IBD segment. Len.accuracy is the proportion of the maximum lengths overlapped between the ground-truth and reported IBD segments divided by the reported lengths. See Tang et al. [43] for more details.

The results showed that clusIBD performed similarly to IBIS and TRUFFLE but worse than IBDseq for detecting IBD segments smaller than 7 Mb (Figure S3). As a significant proportion of the short segments identified were false positives (Figure S4), as previously reported [29,31,44], segments smaller than 7 Mb were excluded for all four methods. **Figure 2** shows the performance of clusIBD and the three alternative methods (IBIS, TRUFFLE, and IBDseq) for the detection of IBD segments. The four methods performed similarly when the genotyping error rate was low. However, clusIBD was much more robust when the genotyping error rate exceeded 1%. For example, at an error rate of 5%, approximately 85% of the 10-Mb segments were recalled by clusIBD, while the recall rates for the other three methods were all less than 10%. clusIBD appears to have greater power to detect long IBD segments than short ones. When the genotyping error rate exceeded 10%, IBIS, TRUFFLE, and IBDseq recalled almost no segments. Notably, clusIBD was still able to detect many IBD segments at such high error rates. Furthermore, when we used a larger number of SNPs per window, more segments could be detected with higher accuracy but at the cost of decreasing call rates for short segments (Figure S5).

In terms of the accuracy of IBD segments detected, clusIBD, IBDseq, and TRUFFLE performed similarly, achieving slightly higher accuracy than IBIS (Figure 2, Figure S6). IBDseq performed the best among the four methods, with almost 100% accuracy in detecting IBD segments of different lengths when the genotyping error rate was no more than 5% (Figure S6). However, when the error rate exceeded 5%, IBDseq could only detect one or two segments for each length group. Most of these were marginally larger than the predefined threshold, and some of which were incorrect. In contrast, clusIBD was able to detect many more IBD segments with consistent accuracy irrespective of the level of genotyping error in the genetic data. When the lengths were taken into consideration for accuracy estimation (*i.e.*, len.accuracy), IBDseq performed the best, while clusIBD, IBIS, and TRUFFLE showed similar len.accuracy (Figure 2, Figure S6). Owing to random matches at SNP sites of non-IBD segments, clusIBD (also IBIS and TRUFFLE) tended to underestimate the start positions and overestimate the end ones (Figure S7). Nevertheless, the median difference between estimated and actual breakpoints for clusIBD was less than 0.5 Mb and the difference decreased with increasing genotyping error rates, resulting in a slightly higher len.accuracy (see Figure S7 and Table S1 for details).

We further investigated whether the detection of IBD segments varied depending on the relationships and the population of origin of the samples. The ground-truth IBD segments of simulated first-to-seventh-degree relationships were categorized into three segment size bins: [7 Mb, 15 Mb), [15 Mb, 25 Mb), and [25 Mb, 35 Mb), and segments larger than 35 Mb were not included. The results showed that there was a slight but consistent decrease in recall and power to detect IBD segments from the distant relationships, which was more evident for shorter segments and exacerbated by higher genotyping error rates (Figure S8). Surprisingly, slight decreases in recall and accuracy were also observed for first-degree relationships. This can be explained by the fact that some adjacent ground-true but short segments were merged into one long segment by clusIBD. As a result, the reported segments were not considered correct because they were not covered by ≥ 50% by any ground-truth IBD segment. In addition, clusIBD performed similarly for samples from different populations, except for a slight decrease for those of Mexican ancestry from Los Angeles (Figure S9).

### Kinship inference of simulated data

We then evaluated the performance of clusIBD in classifying relationships ranging from first- to seventh-degree relationships, as well as unrelated individuals using simulated family data based on the 1000 Genomes Project dataset. IBDseq only reports IBD segments, regardless of whether they are IBD1 or IBD2. Since a pair of full siblings is expected to share 25%, 50%, and 25% of IBD2, IBD1, and non-IBD segments, respectively, thus two-thirds of these reported segments are expected to be IBD1 and one-third to be IBD2. Therefore, we estimated the total length for IBD1 by multiplying the reported IBD segments by 2/3 and for IBD2 by 1/3 for a full sibling pair. Kinship coefficient estimation and relationship classification were performed as described in the Method section.

**Figure 3:**
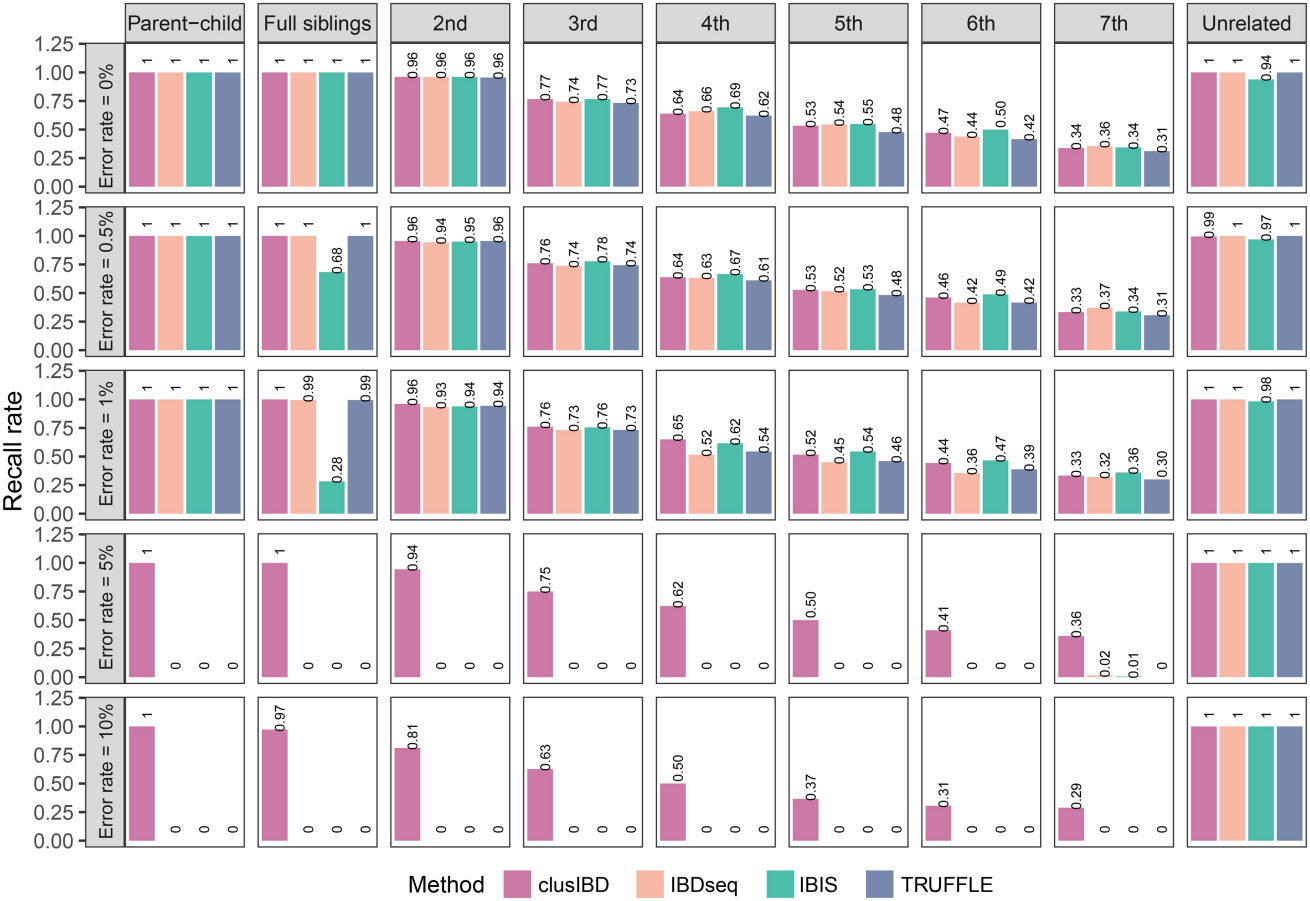
Comparison of clusIBD, IBDseq, IBIS, and TRUFFLE in kinship inference at different levels of genotyping error Recall rate is the proportion of cases in which a relationship is correctly assigned as the true relationship.

In the absence of genotyping error, the agreement of kinship coefficients between clusIBD and the other three methods (IBIS, TRUFFLE, and IBDseq) was very high, with *r*^2^ values of 0.999, 0.999, and 0.997, respectively (Figure S10). IBIS tended to slightly underestimate the kinship coefficients for full siblings compared with the other three algorithms at an error rate of 1%. After reviewing the results of IBIS, we found that many IBD2 segments were misclassified as IBD1 segments, which explains this discrepancy. Furthermore, *r*^2^ values dropped dramatically at an error rate of 5%, and kinship coefficients were all largely underestimated with IBIS, TRUFFLE, and IBDseq. In contrast, clusIBD could robustly estimate kinship coefficients at an error rate of 10%. In terms of relationship classification, all algorithms performed well and had comparable recall rates for inferring close and distant relationships from genetic data with no genotyping error (**Figure 3**). clusIBD outperformed IBIS, TRUFFLE, and IBDseq when the genotyping error rate was 1% or higher. In particular, when the three alternative methods had near-zero recall rates at a 5% error rate for all the relationships, clusIBD still successfully recalled them with high accuracy. IBIS had low power to identify full siblings even at a low level of genotyping error (1%), which can be attributed to its inability to identify IBD2 segments in the presence of genotyping error. These results demonstrate that clusIBD can robustly infer a relationship from genetic data with varying levels of genotyping error and outperforms the three alternative methods in this regard.

### Kinship inference of poor-quality DNA

DNA from six individuals from a multigeneration family (Figure S2) was collected with informed consent (Human Subject IRB Protocol identifier: [2023]016, approved by the Ethics Committee of Zhongshan School of Medicine, Sun Yat-sen University, China). The DNA was then diluted and degraded to mimic low-quantity and low-quality samples. These samples were finally genotyped using the Infinium ASA (Illumina). After quality control and SNP filtering (see Method), approximately 300,000 SNPs were retained. Prior to IBD detection, we evaluated the level of genotyping error for these artificial poor-quality DNA samples. The group with 100 ng of undegraded DNA was treated as a reference, and three types of genotyping error (drop-in, dropout, and opposite-homozygote errors) were estimated for the remaining samples. Drop-in error refers to a homozygote being reported as a heterozygote, and *vice versa* for dropout error. Opposite-homozygote error refers to a scenario in which different homozygotes are reported. As shown in Figure S11 and Table S2, although the input of 200 ng of high-quality DNA is recommended by the manufacturer of the ASA, we still found low genotyping error rates (< 0.5%) for as little as 0.5 ng of DNA or DNA with average fragment size of approximately 800 bp. However, when the DNA input was very low (0.1 ng) or highly degraded (with average fragment size of approximately 150 bp and 400 bp), genotyping errors increased significantly. Specifically, the average error rates were 4.30% ± 2.36%, 12.49% ± 1.24%, and 0.84% ± 0.26% for the 0.1 ng, 150 bp, and 400 bp groups, respectively. Moreover, the pattern of genotyping error appeared to differ among the different groups. Specifically, the predominant error was allele dropout for the low-quantity group (*i.e.*, 0.1 ng group), whereas it was allele drop-in for the low-quality groups (*i.e.*, 150 bp and 400 bp groups). Meanwhile, the rates of opposite-homozygote error were extremely low for all groups (Figure S11; Table S2).

**Figure 4:**
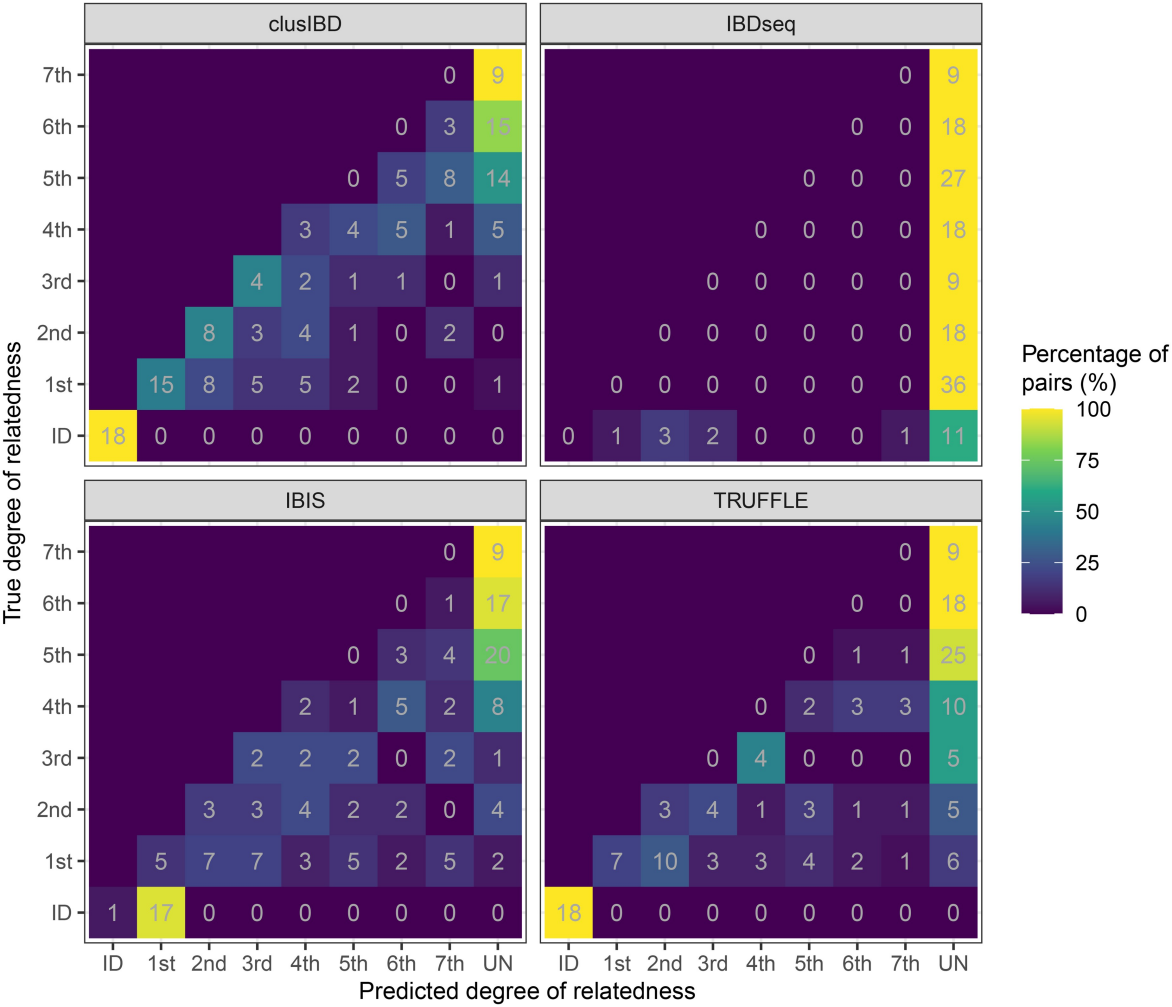
Comparison of clusIBD, IBDseq, IBIS, and TRUFFLE in kinship inference for poor-quality DNA samples Eighteen samples from the 0.1 ng, 150 bp, and 400 bp groups were included. First- to seventh-degree relationships were assigned for a total of 36, 18, 9, 18, 27, 18, and 9 pairs, respectively, as well as 18 pairs of identical samples. ID, identical sample; UN, unrelated individual.

For kinship inference, our focus was mainly on the low-quantity and low-quality groups (the 0.1 ng, 150 bp, and 400 bp groups). Among the 18 samples (6 samples for each group), there were 36, 18, 9, 18, 27, 18, and 9 pairs of first- to seventh-degree relationships, respectively, as well as 18 pairs of identical samples (**Figure 4**, Figure S2). We then used the four tools to detect IBD segments in the dataset, and the relationship was determined as described above. Overall, clusIBD achieved the highest accuracy (31.37%), followed by TRUFFLE (18.30%), IBIS (8.50%), and IBDseq (0%). IBDseq detects IBD segments based on IBD LOD (logarithm of the odds) score, which is calculated using the variant’s MAF. It also requires linkage disequilibrium (LD)-based thinning of the variants so that no pair of variants is strongly correlated [30]. However, all the samples in this dataset were related to each other, and the MAF estimation and LD-based thinning cannot be handled correctly, which may explain the poor performance of IBDseq. The recall rates for each type of relationship were 100% (18/18), 41.67% (15/36), 44.44% (8/18), 44.44% (4/9), 16.67% (3/18), 0%, 0%, and 0% for identical individuals and first- to seventh-degree relationships, respectively, all of which were higher than the corresponding rates for the other three methods. These values increased to 100%, 63.89%, 61.11%, 66.67%, 38.89%, 18.52%, 16.67%, and 0%, respectively, when the predicted degree of relatedness was considered to be correct if it was within one degree of the actual one. Although the nine pairs of seventh-degree relationships were all incorrectly assigned as unrelated, a long IBD segment of 16–22 Mb was identified on chromosome 1 for six of these nine pairs (Table S3), strongly suggesting their relatedness.

### Kinship inference of ancient DNA

**Figure 5:**
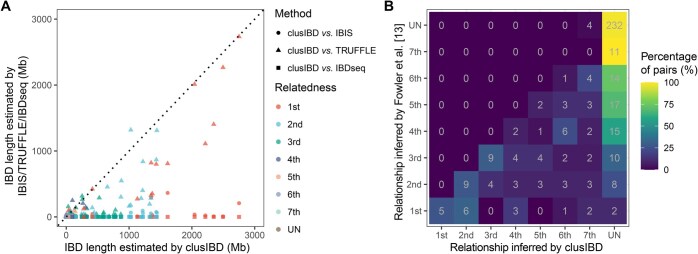
IBD segment length estimation and kinship inference for ancient DNA samples **A**. Scatter plot showing IBD segment lengths estimated by clusIBD *vs.* IBIS, TRUFFLE, and IBDseq. Dotted line represents a line with a slope of 1, *i.e.*, y = x, and points below the line indicate greater IBD lengths estimated by clusIBD than by IBIS (circle), TRUFFLE (triangle), and IBDseq (square). **B**. Matrix of the matches of relationships inferred by Fowler et al. [13] and by clusIBD. 500 SNPs were used per window. SNP, single nucleotide polymorphism.

Finally, we analyzed the 30 ancient samples from the Hazleton North long cairn. The ancient DNA was sequenced by Fowler et al. [13] using whole-genome capture technology. One sample (SC5m) was removed because the authors of the original study could not position it definitively in the pedigree. As shown in **Figure 5**A, clusIBD reported larger summed IBD lengths than IBIS, TRUFFLE, and IBDseq did for the majority of related individuals, indicating that clusIBD is highly tolerant of genotyping errors. Of the three alternative tools, TRUFFLE performed best, as it incorporates an error model that corrects for segment break-ups due to genotyping errors [31], while IBDseq performed worst. We further inferred the relationships as described in the Method section and compared the results with the pedigree constructed by Fowler and colleagues [13]. With the default settings, the estimated number of SNPs per window was 150. Only one pair of related individuals was correctly assigned, resulting in a concordance rate of 0.50% (Figure S12A and B). This increased to 3.02% with 300 SNPs per window (Figure S12C and D), and 14.07% with 500 SNPs per window (Figure 5B), approximately 6 and 30 times higher than with the default settings, respectively.

Interestingly, although the relatedness was underestimated for most of these individuals, four pairs that were once recognized as unrelated in the study by Fowler et al. [13] were inferred by clusIBD as seventh-degree relationships. The four pairs involved four family members and two unrelated individuals. NE4m, one of the five unrelated males as inferred by Fowler and colleagues, shared a long segment (> 30 Mb) with three family members: SC2m, SP1m, and SE1m (Table S4). SC2m and SP1m were full siblings and shared a segment of approximately 36 Mb with NE4m at almost the same positions (Chr3: 80–118 Mb). NE4m also shared a 32-Mb IBD segment at Chr14: 51–83 Mb with SE1m, who was the offspring of a distant relative of the core family member NC1m. Considering that the burial of a nonpaternal male in the tomb was rare, we speculated that NE4m was a distant relative of NC1m. Similarly, NC10m also shared a long segment with two family members (SP3m and NC7f, who were also full siblings; Table S4) and could thus be distantly related to the family. In summary, clusIBD not only successfully recovered some of the related individuals from the 30 ancient samples but also identified two additional males as being related to the family. Since only three males in the grave were unrelated to the family, we may conclude that it was rare in this period for kinship to include social ties independent of biological relatedness.

### Computation time

Five datasets, each comprising 100, 200, 300, 400, and 500 individuals, were simulated based on our established reference panels. The computation times of clusIBD, IBIS, TRUFFLE and IBDseq were then compared. Since IBDseq only analyzes one chromosome at a time, its computation time was estimated by simply summing the time over 22 chromosomes. All four tools allow parallel computation with a specified number of cores, and the times reported here are those using an Intel^®^ Xeon^®^ CPU E5-2650 v4 @ 2.20 GHz processor with five cores. The computation time for clusIBD was 47 s for the 100-sample batch, and the time increased quadratically with increasing sample size (Figure S13). For batches of 500 samples, the average time cost was about 0.03 s per core per pair of samples. IBIS was the most computationally efficient algorithm, taking only about 2 s for 100 samples. In contrast, IBDseq was the least efficient, taking more than 200 s for 100 samples. The most time-consuming process for IBDseq was LD-based thinning. Overall, clusIBD was faster than IBDseq but slower than IBIS and TRUFFLE.

## Discussion

In this study, we propose clusIBD, for detecting IBD segments from unphased genetic data with different levels of genotyping error. We have compared clusIBD with several existing IBD detection methods using simulated family data and real SNP array and whole-genome sequencing data. Our results demonstrated that clusIBD can robustly detect IBD segments from materials containing DNA of varying quality and quantity, and may be a promising tool for forensic genetics and ancient DNA analysis.

One of the key advantages of our method is its robustness to genotyping error. Existing IBD detection methods are fast and accurate when applied to samples with high-quality and high-quantity DNA, but they only allow for relatively low levels of genotyping error (0.1%–1% by default) [29–31]. Many materials, such as ancient DNA, crime scene samples, and noninvasively collected tissues [36,37], are prone to genotyping error, typically having frequencies above this limit. This makes them incapable of detecting IBD segments from such samples. In contrast, clusIBD works by setting a threshold to distinguish IBD segments from non-IBD ones based on difference in R_oph_ (the rate of opposite homozygous genotypes) distributions. As clusIBD can adjust the threshold dynamically based on the level of genotyping error for each sample pair, it can robustly detect IBD segments from materials containing DNA of varying quality and quantity. In addition, for extremely challenging materials, users can further improve the robustness by increasing the number of SNPs per window, although this reduces the power to identify short IBD segments (Figure S5). IBIS also allows users to increase the acceptable error rate in a segment before it is considered false. This feature is intended to facilitate the identification of more IBD segments in case of genotyping error. However, IBIS is not an error-aware algorithm, and increasing the threshold will result in a reduction in accuracy (Figure S14). Turner et al. [28] showed that there was no combination of reasonably permissive parameters that could rescue the performance of the IBD detection methods that they studied (*e.g*., IBIS) when the genotyping error was in the range of 1%–5%. Therefore, it may be impractical to simply increase the acceptable error rates, which increases the risk of false positives. Another way of controlling genotyping error is to use a gap-filling and seed-extension strategy. A similar process has also been applied in alternative tools for phased data [26,32]. Freyman et al. [27] showed that some IBD detection methods create short but very closely localized IBD segments, which are, in fact, small parts of a long, single, and true IBD segment and should be joined together. In view of this, some methods attempt to address genotyping error post hoc, *e.g*., ibd-end [45] and IBDkin [16]. All of these processes are very useful in improving the robustness of related methods to genotyping error. Some DTC companies (*e.g*., Family Tree DNA [FTDNA] and 23andMe) and third-party services (*e.g*., GEDmatch) use similar half-identical matching algorithms to detect IBD segments with unphased genotypes [44]. We uploaded the genotype files of the 18 poor-quality DNA samples (0.1 ng, 150 bp, and 400 bp groups) to GEDmatch, a well-known third-party service and the main entry point for law enforcement to date. We found that clusIBD recalled longer summed lengths of IBD segments for most of the sample pairs than GEDmatch did, thus outperforming it (Figure S15).

Recent methodological advances have made it possible to perform haplotype phasing and IBD detection on low-quality and/or low-coverage data [32,46,47]. An excellent approach for this involves the use of the combination of GLIMPSE [47] and ancIBD [32], which has been successfully applied to estimate relatedness in free-ranging macaques [48] and to learn demographic histories [49]. Inspired by this, we applied GLIMPSE + clusIBD to analyze the 35 ancient DNA samples from the Hazleton North long cairn. Our results revealed a significant improvement in the accuracy of kinship inference, rising from the initial 0.50% (Figure S12B) to 65.71% (Figure S16), suggesting that combining clusIBD with phasing and imputation (*e.g*., via GLIMPSE) can significantly enhance accuracy. A comprehensive study of such combinatorial approaches will be conducted in the future.

The pseudohaploid-based approach is also computationally efficient and robust to genotyping error, and has been widely applied to the analysis of ancient DNA [50]. Pseudohaploid genotype calling allows for genetic information to be obtained at a nucleotide site covered by only a single read. Several tools using this approach have been developed [22,51,52], which successfully performed kinship inference with shotgun sequencing data with coverage as low as 0.1× [22]. However, these methods either require a dataset adequate to provide a reliable normalization value or require the user to provide a set of allele frequencies. This can be challenging for unknown forensic human remains, archaeological samples, and small conservation populations [53]. Furthermore, if the allele frequencies used are not representative of the target population, there will be significant overrepresentation of the relatedness [54]. In addition, similar to the frequency-based approaches, pseudohaploid-based approaches can only accurately infer close relationships and have high false-positive rates in identifying distant relatives.

For relationship classification, IBD segment-based methods have high accuracy and can identify very distant relationships [23]. It is also possible to discriminate relationships of the same degree of relatedness based on different IBD signals [55,56]. Qiao et al. showed that specific relationship types could be uncovered via multiway IBD sharing, and they were able to classify pairs as maternally or paternally related using a sex-specific genetic map [57]. In addition to kinship inference, IBD segments are also useful for a variety of analyses, including studies of population history [58], estimating mutation rates [59] and recombination rates [60], mapping disease genes [61–64], and identifying recent positive selection [45,65].

Our method has some potential limitations that should be mentioned here. For example, clusIBD has reduced sensitivity with regard to identifying short IBD segments. This may be acceptable for certain analyses because closely related individuals are expected to share long IBD segments. In addition, there is potential to overcome this by allowing for more SNP sites to be analyzed. Another limitation of our approach is the inability to infer IBD segments on the X chromosome, which is useful to study sex-specific migration patterns [66].

## Conclusion

In summary, clusIBD can robustly detect IBD segments from materials containing DNA of varying quality and quantity, irrespective of whether the data are from an SNP array or whole-genome sequencing. It can be used to accurately infer relationships from unphased genetic data with high genotyping error rates (∼ 10%) and is a promising tool for forensic genetics and ancient DNA analysis.

## Ethical statement

Written informed consent was obtained from the participating subjects, and this study was approved by the Ethics Committee of Zhongshan School of Medicine, Sun Yat-sen University, Guangzhou, China (Approval No. [2023]016).

## Code availability

ClusIBD is publicly available at GitHub (https://github.com/Ryan620/clusIBD/). The code has also been submitted to BioCode at the National Genomics Data Center (NGDC), China National Center for Bioinformation (CNCB) (BioCode: BT007882), which is publicly accessible at https://ngdc.cncb.ac.cn/biocode/tool/BT007882. All the source codes for data analysis and figure generation are also available in the GitHub repository (https://github.com/Ryan620/clusIBD/tree/main/source_code).

## Supplementary Material

qzaf055_Supplementary_Data

## Data Availability

The genetic data of the six modern family members have been deposited in the Genome Variation Map at the NGDC, CNCB (GVW: GVM000992), which are publicly accessible at https://ngdc.cncb.ac.cn/gvm/getProjectDetail?project=GVM000992.

## CRediT author statement

**Ran Li:** Conceptualization, Methodology, Software, Writing – original draft, Funding acquisition. **Yu Zang:** Investigation, Formal analysis, Data curation, Writing – review & editing. **Zhentang Liu:** Methodology, Software. **Jingyi Yang:** Investigation, Validation. **Nana Wang:** Visualization, Validation. **Jiajun Liu:** Visualization. **Enlin Wu:** Investigation. **Riga Wu:** Supervision, Project administration. **Hongyu Sun:** Conceptualization, Writing – review & editing, Project administration, Funding acquisition. All authors have read and approved the final manuscript.
