# Supplementary material for: clusIBD: Robust Detection of Identity-by-descent Segments Using Unphased Genetic Data from Poor-quality Samples
Source: Genomics Proteomics Bioinformatics. 2025 Jun 20;23(3):qzaf055. doi: 10.1093/gpbjnl/qzaf055 (PMC12449261; doi:10.1093/gpbjnl/qzaf055)
Supplement: qzaf055_Supplementary_Data [file qzaf055_supplementary_data.zip › supplementary material captions.docx]

**Supplementary materials**

**Figure S1 Distributions of the rates of opposite homozygous genotypes for different relationships**

In this example, we show a scenario where one person has an error rate of 10%, while many others (including the relatives of the person of interest) in the dataset have an error of 0.5%. Bimodal peaks are observed for 1^st^ – 3^rd^ degree relationships, the smaller of which (*i.e.* near the y-axis) corresponds to windows of IBD segments and the larger of which corresponds to non-IBD segments. The three metrices, *i.e.*, t_universal_, t_valley_ and t_15th_, are estimated by clusIBD and the final threshold is the maximum of them. For close relationships, t_valley_ is greater than t_universal_ and t_15th_, and so would be the threshold. However, for distant (4^th^ degree or more distant) relationships, null results are obtained for t_valley_, and t_universal_ is greater than t_15th_, so t_universal_ is chosen. Sometimes, t_15th_ is greater than t_universal_ for very distant relationships and unrelated individuals, and t_15th_ is used as the threshold. Since the threshold can be adapted to different levels of genotype error for the sample pair of interest, clusIBD is always able to detect IBD segments at its best. IBD, identity by descent.

**Figure S2** **The pedigree of the studied family**

Samples filled in blue were collected.

**Figure S3 Performance of clusIBD, IBIS, TRUFFLE, and IBDseq for detecting IBD segments ranging from 4 Mb to 7 Mb**

A total of five hundred artificial IBD segments are analyzed for each length group and no genotyping errors are introduced. IBD, identity by descent; Mb, megabases.

**Figure S4 Performance of clusIBD for detecting IBD segments ranging from 2 Mb to 15 Mb**

A total of five hundred artificial IBD segments are analyzed for each length group and no genotyping errors are introduced. IBD, identity by descent; Mb, megabases.

**Figure S5 Performance of clusIBD with different numbers of SNPs per window**

A total of five hundred artificial IBD segments are analyzed for each length and error group. IBD segments of 20 Mb in length are used for parts **A**, **B**, and **C**. SNPs, single nucleotide polymorphisms; IBD, identity by descent; Mb, megabases.

**Figure S6 Performance of clusIBD, IBIS, TRUFFLE, and IBDseq for detecting IBD segments (zooming in on the 0.75–1.00 y-axis region)**

Accuracy is the proportion of reported IBD segments that are covered by any one ground-truth IBD segment by $\geq$50%. Len.accuracy is the proportion of the maximum lengths overlapped between the ground-truth and detected IBD segment divided by the reported lengths. See Tang et al, Gigascience, 2022 [43] for details. IBD, identity by descent; Mb, megabases.

**Figure S7** **The distribution of the difference between estimated and actual breakpoints by clusIBD**

A negative value indicates an underestimated position and a positive value indicates an overestimated position. Mb, megabases.

**Figure S8** **Performance of clusIBD for detecting IBD segments from different relationships**

The ground truth IBD segments for 1^st^ to 7^th^ degree relationships are categorized into three segment size bins: [7 Mb, 15 Mb), [15 Mb, 25 Mb) and [25 Mb, 35 Mb), and segments larger than 35 Mb were not included. IBD, identity by descent; Mb, megabases.

**Figure S9 Performance of clusIBD for detecting IBD segments between samples from different populations**

CEU, Utah residents with north and west European ancestry; CHB, Han Chinese in Beijing; GIH, Gujarati Indian from Houston; MXL, Mexican ancestry from Los Angeles; and YRI, Yoruba in Ibadan. IBD, identity by descent; Mb, megabases.

**Figure S10 Scatter plots of kinship coefficients (**$\boldsymbol{\theta}$**) estimated by clusIBD and IBIS, TRUFFLE, and IBDseq**

The $\theta$ values are estimated using the formula: $\theta=\frac{L(IBD2)}{2\times L(genome)}+\frac{L(IBD1)}{4\times L(genome)}$, where $L(IBD1)$, $L(IBD2)$, and $L(genome)$ are the total lengths of IBD1 segments, IBD2 segments, and the whole genome, respectively. Since IBDseq does not distinguish between IBD1 and IBD2 segments, we estimated the total length of IBD1 by multiplying the reported IBD by 2/3 and for IBD2 by 1/3 for a full sibling pair. The dotted black lines represent a line with a slope of 1, *i.e.*, y=x. IBD, identity by descent; IBD1, IBD in one copy of the genome; IBD2, IBD in both copies of the genome.

**Figure S11** **The rates of genotype errors of the artificial poor-quality DNA**

Drop-in, a homozygote is reported as a heterozygote; dropout, a heterozygote is reported as a homozygote; opposite-homozygote error, opposite homozygotes are reported; all, the sums of the three types of genotype errors; ng, nanograms; bp, base pairs.

**Figure S12 IBD lengths and kinship inference for ancient DNA samples**

**A.** and **C.** Scatter plots of IBD lengths estimated by clusIBD and IBIS, TRUFFLE, and IBDseq. Dotted lines represent a line with a slope of 1, *i.e.*, y=x, and points below the line indicate a larger IBD lengths by clusIBD than by IBIS (circle), TRUFFLE (triangle), and IBDseq (square). **B.** and **D.** Match matrices of inferred relationships by Fowler et al. and by clusIBD. Different parameters were used, with the default setting for parts A and B, and 300 SNPs per window for parts C and D. IBD, identity by descent; UN, unrelated individuals; Mb, megabases.

**Figure S13 Comparison of the running time of clusIBD, IBIS, TRUFFLE, and IBDseq**

All the analyses are performed on an Intel(R) Xeon(R) CPU E5-2650 v4 @ 2.20GHz processor using five cores.

**Figure S14 Performance of IBIS when using different acceptable errors**

The figure is generated based on five hundred of simulated 20 Mb segments. Mb, megabases.

**Figure 15 Comparison of clusIBD and GEDmatch method for detecting IBD segments for the eighteen artificial poor-quality DNA**

Black dotted lines represent a line with a slope of 1, *i.e.*, y=x, and points below the line indicate a larger IBD lengths (Mb) by clusIBD than by GEDmatch. 150 (default), 200, and 250 SNPs per window were used for parts **A**, **B**, and **C**, respectively. SNPs, single nucleotide polymorphisms; Mb, megabases.

**Figure S16 Match matrices of inferred relationships by Fowler et al. and by GLIMPSE + clusIBD**

The genetic data of the thirty-five ancient samples were phased and imputed using GLIMPSE [47] and the 1000 Genome haplotype reference panel. After applying quality control (QC), eight million SNPs were retained and all samples passed the QC thresholds. IBD segments were then detected using clusIBD with the default settings. IBD, identical by descent; UN, unrelated individuals.

**Table S1 The median difference (Mb) between estimated and actual breakpoints by clusIBD**

**Table S2 The types and rates of genotype error of the artificial poor-quality DNA samples**

**Table S3 IBD segments identified by clusIBD for the artificial poor-quality DNA samples**

**Table S4 IBD segments identified by clusIBD for the ancient DNA samples**
