# Supplementary material for: clusIBD: Robust Detection of Identity-by-descent Segments Using Unphased Genetic Data from Poor-quality Samples
Source: Genomics Proteomics Bioinformatics. 2025 Jun 20;23(3):qzaf055. doi: 10.1093/gpbjnl/qzaf055 (PMC12449261; doi:10.1093/gpbjnl/qzaf055)
Supplement: qzaf055_Supplementary_Data [file qzaf055_supplementary_data.zip › Table S1.docx]

**Table S1 The median difference between estimated and actual breakpoints by clusIBD**

| **Error rates** | **Start (Mb)** | **End (Mb)** |
| --- | --- | --- |
| 0 | -0.300 | 0.361 |
| 0.01 | -0.278 | 0.256 |
| 0.05 | -0.070 | 0.063 |
| 0.1 | 0.324 | −0.338 |

*Note*: A negative value indicates an underestimated position, *i.e*., the estimated position is smaller than the true breakpoint. A positive value indicates an overestimated position. Mb, megabases.
