# Supplementary material for: clusIBD: Robust Detection of Identity-by-descent Segments Using Unphased Genetic Data from Poor-quality Samples
Source: Genomics Proteomics Bioinformatics. 2025 Jun 20;23(3):qzaf055. doi: 10.1093/gpbjnl/qzaf055 (PMC12449261; doi:10.1093/gpbjnl/qzaf055)
Supplement: qzaf055_Supplementary_Data [file qzaf055_supplementary_data.zip › Table S2.docx]

**Table S2 The types and rates of genotype error of the artificial poor-quality DNA samples**

| **Reference** | **Samples** | **Sample types** | **All** | **Drop-in error** | **Dropout error** | **Opposite-homozygote error** |
| --- | --- | --- | --- | --- | --- | --- |
| A01_100ng | A01_10ng | 10ng | 4.20E-04 | 3.79E-04 | 3.36E-05 | 6.71E-06 |
| A01_100ng | A01_1ng | 1ng | 9.44E-04 | 8.66E-04 | 6.09E-05 | 1.69E-05 |
| A01_100ng | A01_0.5ng | 0.5ng | 2.53E-03 | 2.26E-03 | 2.61E-04 | 1.37E-05 |
| A01_100ng | A01_0.1ng | 0.1ng | 5.14E-03 | 3.68E-03 | 1.45E-03 | 1.07E-05 |
| A01_100ng | A01_1500bp | 1500bp | 2.47E-04 | 1.81E-04 | 5.02E-05 | 1.67E-05 |
| A01_100ng | A01_800bp | 800bp | 1.93E-03 | 1.78E-03 | 1.36E-04 | 1.36E-05 |
| A01_100ng | A01_400bp | 400bp | 5.19E-03 | 4.87E-03 | 3.03E-04 | 1.39E-05 |
| A01_100ng | A01_150bp | 150bp | 1.37E-01 | 8.84E-02 | 4.63E-02 | 2.27E-03 |
| A03_100ng | A03_10ng | 10ng | 1.23E-04 | 9.67E-05 | 2.67E-05 | 0.00E+00 |
| A03_100ng | A03_1ng | 1ng | 3.19E-03 | 2.98E-03 | 2.03E-04 | 1.03E-05 |
| A03_100ng | A03_0.5ng | 0.5ng | 4.60E-03 | 4.07E-03 | 5.23E-04 | 6.98E-06 |
| A03_100ng | A03_0.1ng | 0.1ng | 3.12E-02 | 1.11E-02 | 2.01E-02 | 3.07E-05 |
| A03_100ng | A03_1500bp | 1500bp | 1.56E-04 | 1.03E-04 | 4.99E-05 | 3.33E-06 |
| A03_100ng | A03_800bp | 800bp | 2.50E-03 | 2.36E-03 | 1.33E-04 | 0.00E+00 |
| A03_100ng | A03_400bp | 400bp | 6.21E-03 | 5.91E-03 | 2.83E-04 | 1.75E-05 |
| A03_100ng | A03_150bp | 150bp | 1.34E-01 | 1.06E-01 | 2.68E-02 | 8.94E-04 |
| A20_100ng | A20_10ng | 10ng | 3.78E-04 | 3.25E-04 | 4.02E-05 | 1.34E-05 |
| A20_100ng | A20_1ng | 1ng | 2.67E-03 | 2.52E-03 | 1.38E-04 | 2.07E-05 |
| A20_100ng | A20_0.5ng | 0.5ng | 3.56E-03 | 3.14E-03 | 4.03E-04 | 1.39E-05 |
| A20_100ng | A20_0.1ng | 0.1ng | 4.01E-02 | 1.08E-02 | 2.93E-02 | 4.63E-05 |
| A20_100ng | A20_1500bp | 1500bp | 4.44E-04 | 3.77E-04 | 5.34E-05 | 1.34E-05 |
| A20_100ng | A20_800bp | 800bp | 3.05E-03 | 2.84E-03 | 1.95E-04 | 1.37E-05 |
| A20_100ng | A20_400bp | 400bp | 8.75E-03 | 8.15E-03 | 5.63E-04 | 4.60E-05 |
| A20_100ng | A20_150bp | 150bp | 1.02E-01 | 7.11E-02 | 3.02E-02 | 9.41E-04 |
| D01_100ng | D01_10ng | 10ng | 4.66E-04 | 4.16E-04 | 4.36E-05 | 6.71E-06 |
| D01_100ng | D01_1ng | 1ng | 4.25E-03 | 3.99E-03 | 2.51E-04 | 1.04E-05 |
| D01_100ng | D01_0.5ng | 0.5ng | 5.66E-03 | 5.00E-03 | 6.38E-04 | 2.47E-05 |
| D01_100ng | D01_0.1ng | 0.1ng | 4.68E-02 | 1.49E-02 | 3.18E-02 | 7.47E-05 |
| D01_100ng | D01_1500bp | 1500bp | 2.17E-04 | 1.70E-04 | 4.00E-05 | 6.67E-06 |
| D01_100ng | D01_800bp | 800bp | 3.74E-03 | 3.55E-03 | 1.83E-04 | 3.45E-06 |
| D01_100ng | D01_400bp | 400bp | 1.24E-02 | 1.19E-02 | 4.20E-04 | 3.23E-05 |
| D01_100ng | D01_150bp | 150bp | 1.30E-01 | 1.13E-01 | 1.69E-02 | 6.12E-04 |
| D03_100ng | D03_10ng | 10ng | 1.67E-04 | 1.37E-04 | 2.34E-05 | 6.69E-06 |
| D03_100ng | D03_1ng | 1ng | 1.87E-03 | 1.84E-03 | 3.43E-05 | 0.00E+00 |
| D03_100ng | D03_0.5ng | 0.5ng | 2.14E-03 | 1.92E-03 | 2.06E-04 | 1.37E-05 |
| D03_100ng | D03_0.1ng | 0.1ng | 6.42E-02 | 2.74E-02 | 3.66E-02 | 2.09E-04 |
| D03_100ng | D03_1500bp | 1500bp | 2.14E-04 | 1.50E-04 | 5.68E-05 | 6.68E-06 |
| D03_100ng | D03_800bp | 800bp | 2.62E-03 | 2.49E-03 | 1.27E-04 | 3.42E-06 |
| D03_100ng | D03_400bp | 400bp | 9.69E-03 | 9.21E-03 | 4.71E-04 | 7.14E-06 |
| D03_100ng | D03_150bp | 150bp | 1.22E-01 | 8.95E-02 | 3.16E-02 | 1.14E-03 |
| D10_100ng | D10_10ng | 10ng | 1.90E-04 | 1.67E-04 | 1.34E-05 | 1.00E-05 |
| D10_100ng | D10_1ng | 1ng | 3.65E-03 | 3.48E-03 | 1.56E-04 | 6.95E-06 |
| D10_100ng | D10_0.5ng | 0.5ng | 4.47E-03 | 3.72E-03 | 7.32E-04 | 1.40E-05 |
| D10_100ng | D10_0.1ng | 0.1ng | 7.03E-02 | 2.34E-02 | 4.68E-02 | 1.15E-04 |
| D10_100ng | D10_1500bp | 1500bp | 2.70E-04 | 2.17E-04 | 4.67E-05 | 6.67E-06 |
| D10_100ng | D10_800bp | 800bp | 2.89E-03 | 2.68E-03 | 2.05E-04 | 6.84E-06 |
| D10_100ng | D10_400bp | 400bp | 8.10E-03 | 7.63E-03 | 4.58E-04 | 1.41E-05 |
| D10_100ng | D10_150bp | 150bp | 1.24E-01 | 7.24E-02 | 5.09E-02 | 9.24E-04 |

*Note*: Drop-in error refers to a homozygote being reported as a heterozygote, while dropout refers to a heterozygote being reported as a homozygote; opposite-homozygote error refers to a scenario where different homozygotes are reported. ng, nanograms; bp, base pairs.
