# Supplementary figures and images for: clusIBD: Robust Detection of Identity-by-descent Segments Using Unphased Genetic Data from Poor-quality Samples
Source: Genomics Proteomics Bioinformatics. 2025 Jun 20;23(3):qzaf055. doi: 10.1093/gpbjnl/qzaf055 (PMC12449261; doi:10.1093/gpbjnl/qzaf055)

Density

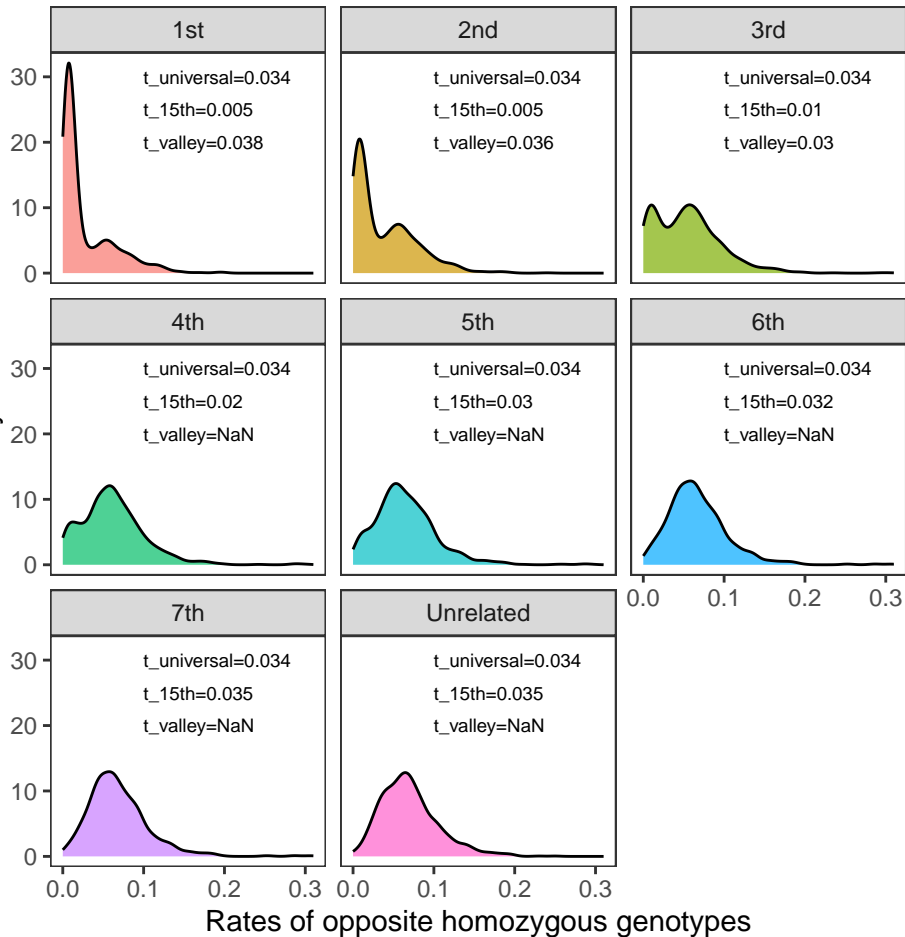

Supplement: qzaf055_Supplementary_Data [file qzaf055_supplementary_data.zip › Figure S1.pdf]

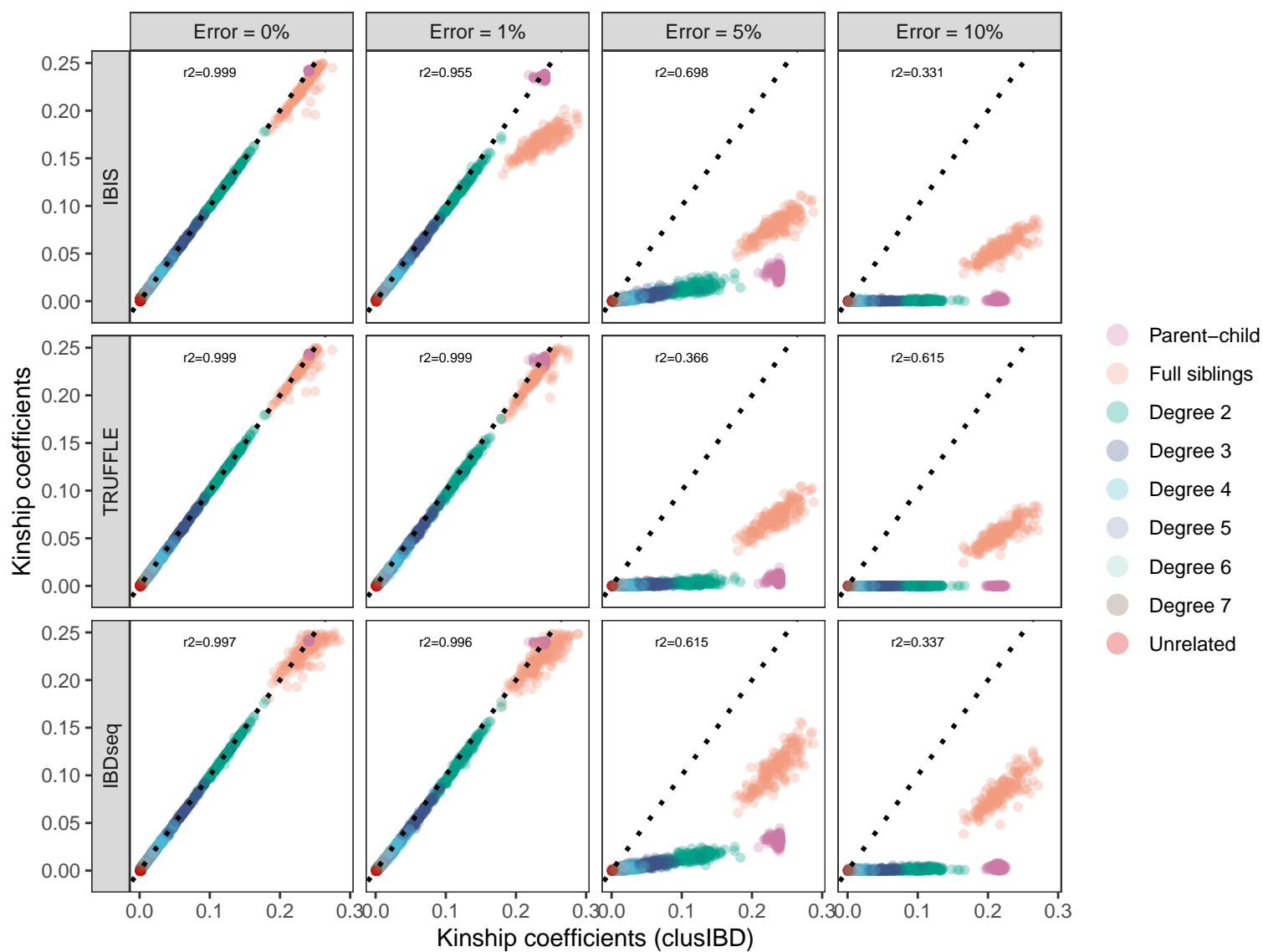

Supplement: qzaf055_Supplementary_Data [file qzaf055_supplementary_data.zip › Figure s10.pdf]

Error rates

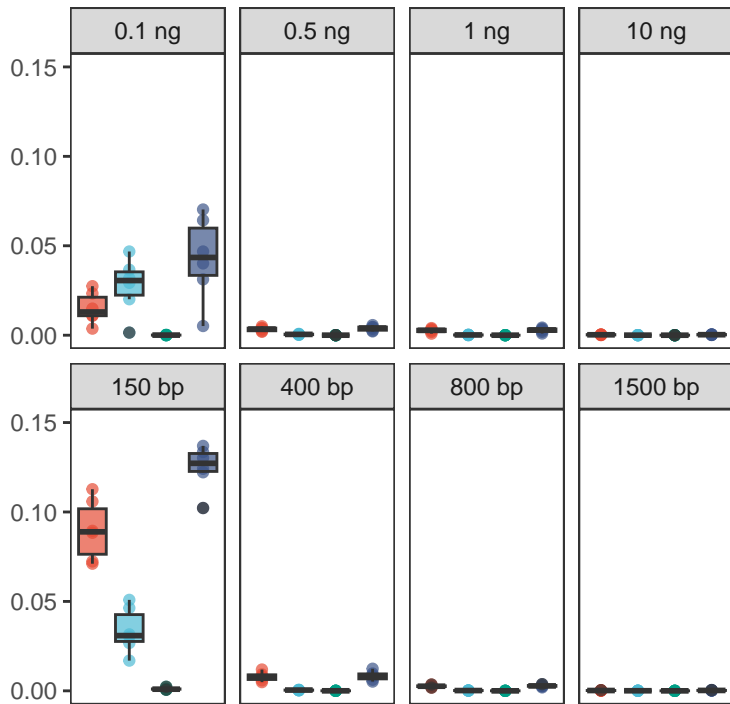

Error types

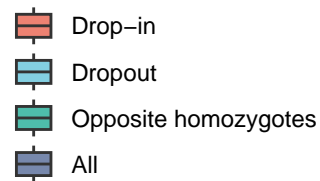

Supplement: qzaf055_Supplementary_Data [file qzaf055_supplementary_data.zip › Figure S11.pdf]

# Methods

- IBIS
- ▲ TRUFFLE
- IBDseq

# Relatedness

- 1st
- 2nd
- 3rd
- 4th
- 5th
- 6th
- 7th
- UN

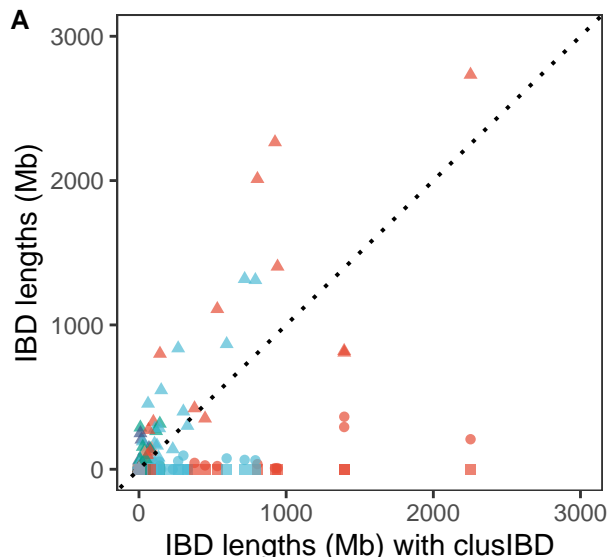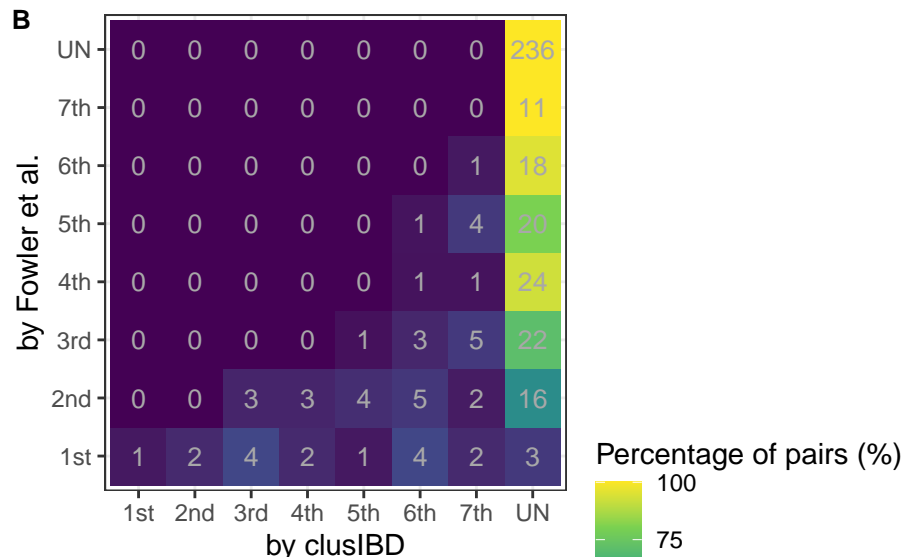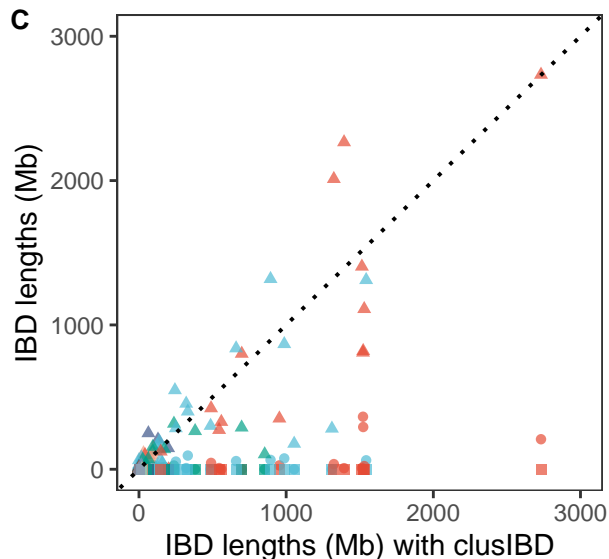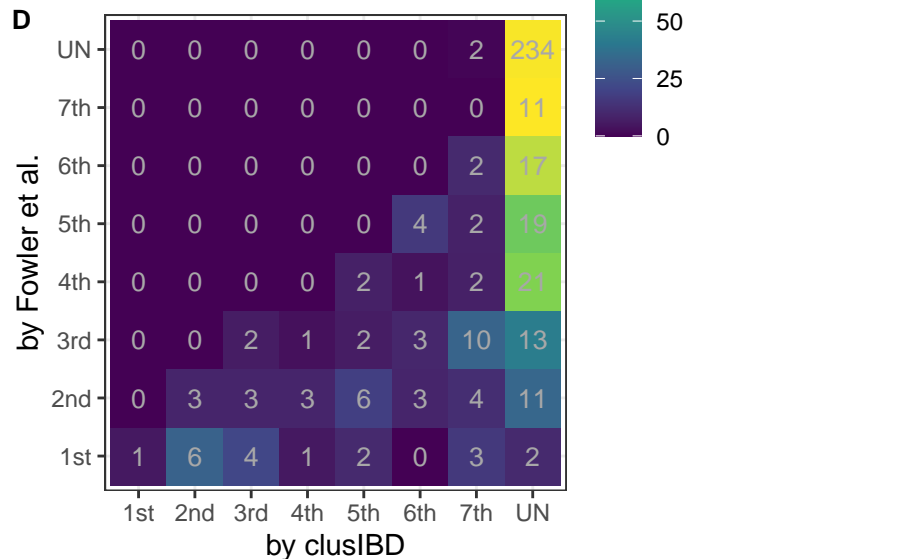

Supplement: qzaf055_Supplementary_Data [file qzaf055_supplementary_data.zip › Figure S12.pdf]

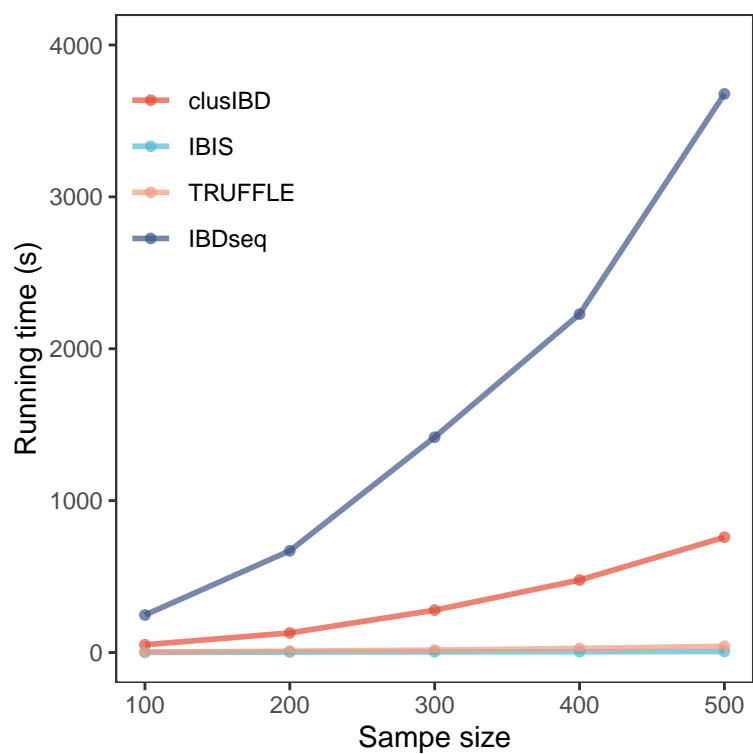

Supplement: qzaf055_Supplementary_Data [file qzaf055_supplementary_data.zip › Figure S13.pdf]

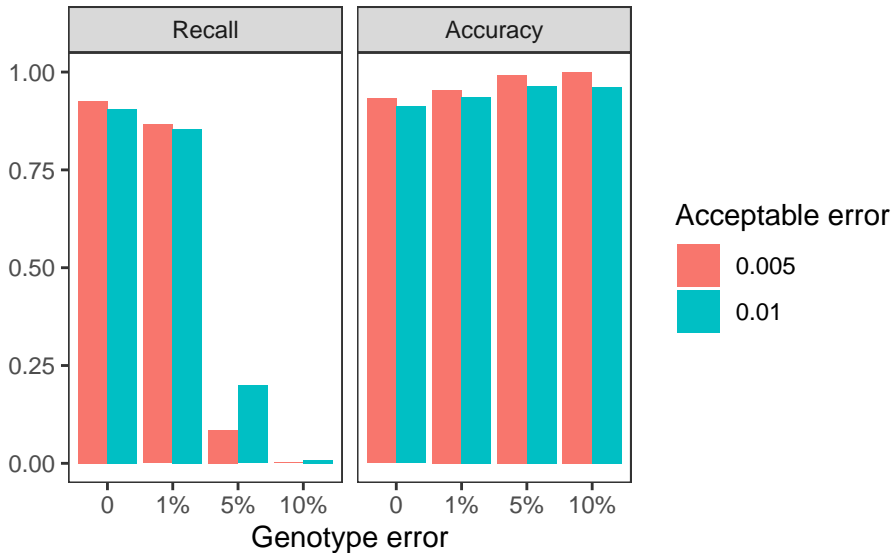

Supplement: qzaf055_Supplementary_Data [file qzaf055_supplementary_data.zip › Figure S14.pdf]

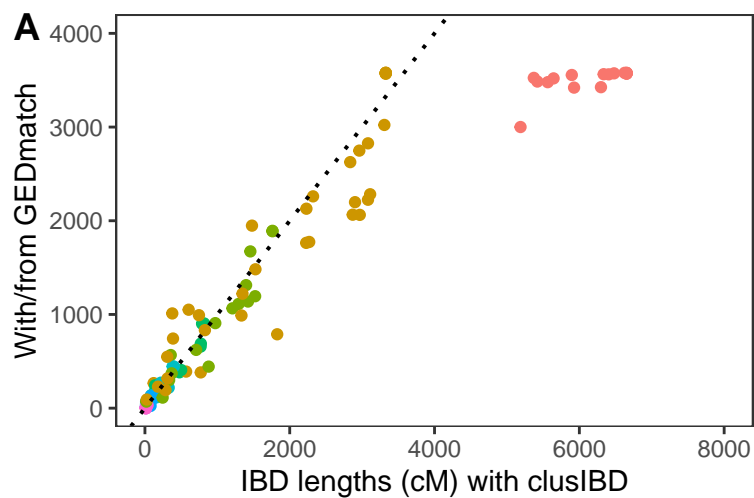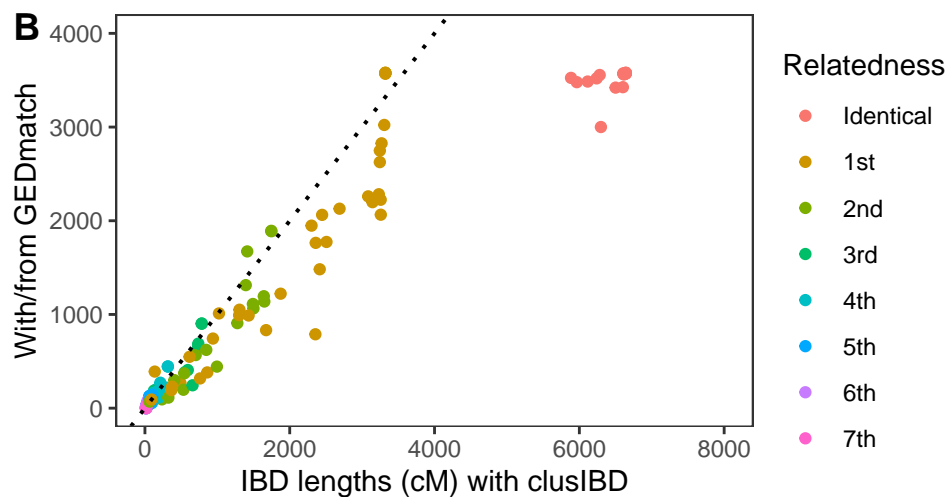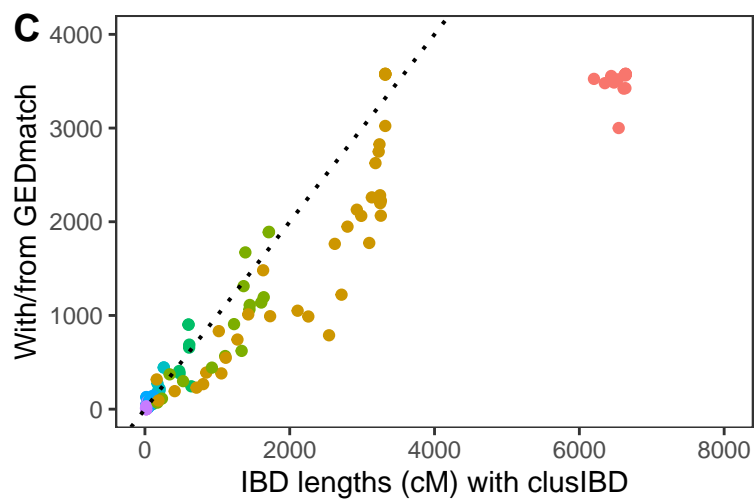

Supplement: qzaf055_Supplementary_Data [file qzaf055_supplementary_data.zip › Figure S15.pdf]

by Fowler et al.

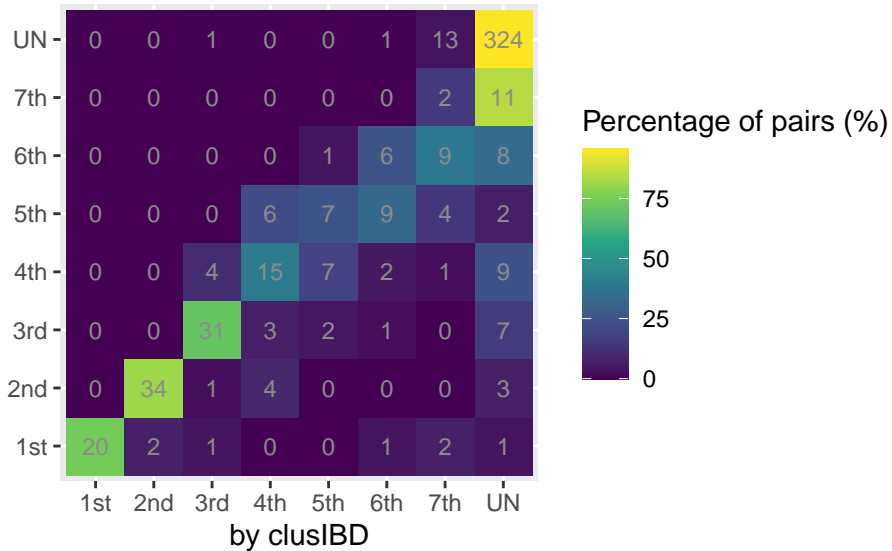

Supplement: qzaf055_Supplementary_Data [file qzaf055_supplementary_data.zip › Figure S16.pdf]

## Slide 1
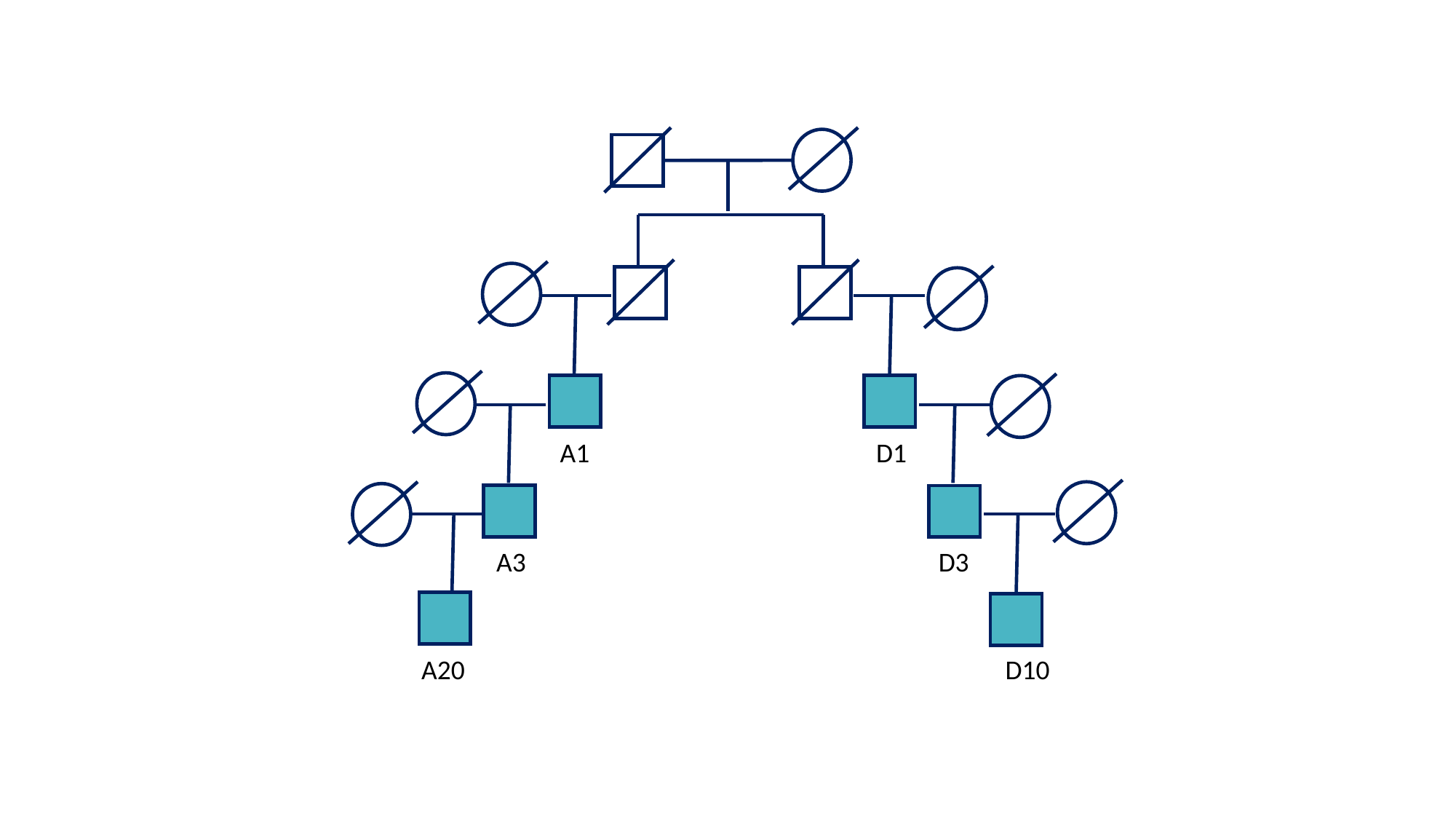

A1
D1
A3
D3
A20
D10

Supplement: qzaf055_Supplementary_Data [file qzaf055_supplementary_data.zip › Figure S2.pptx]

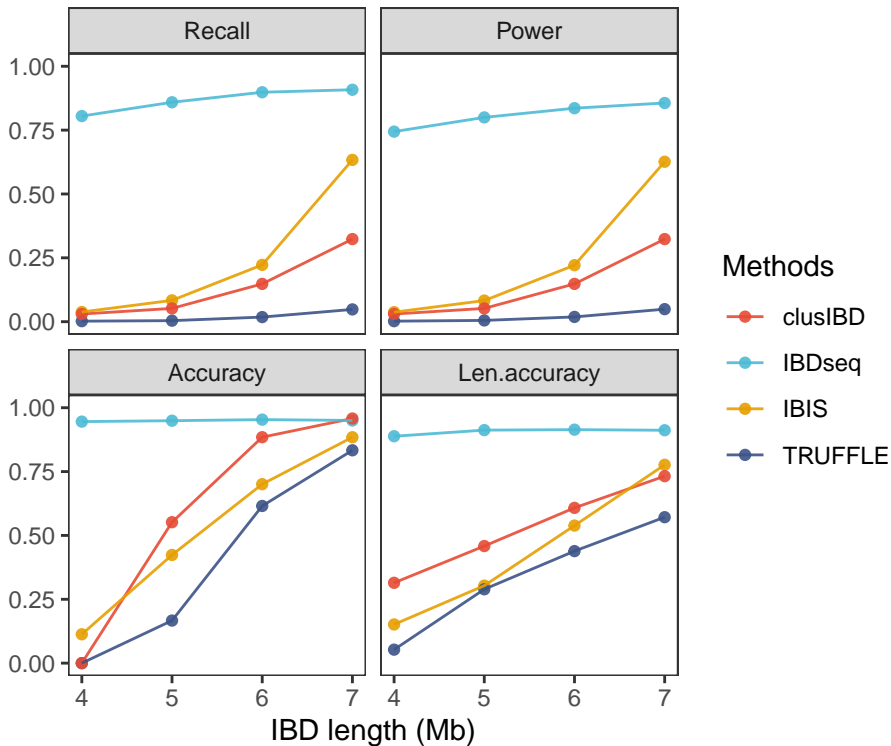

Supplement: qzaf055_Supplementary_Data [file qzaf055_supplementary_data.zip › Figure S3.pdf]

Proportions

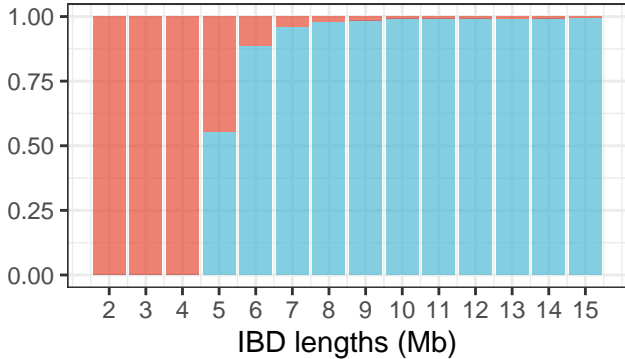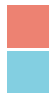

False positives

True positives

Supplement: qzaf055_Supplementary_Data [file qzaf055_supplementary_data.zip › Figure S4.pdf]

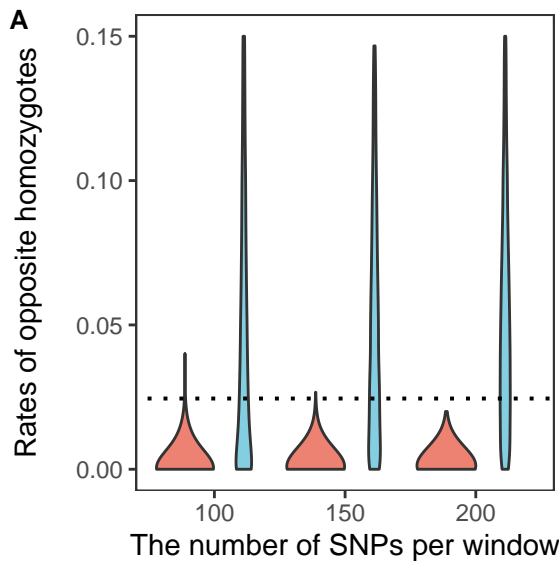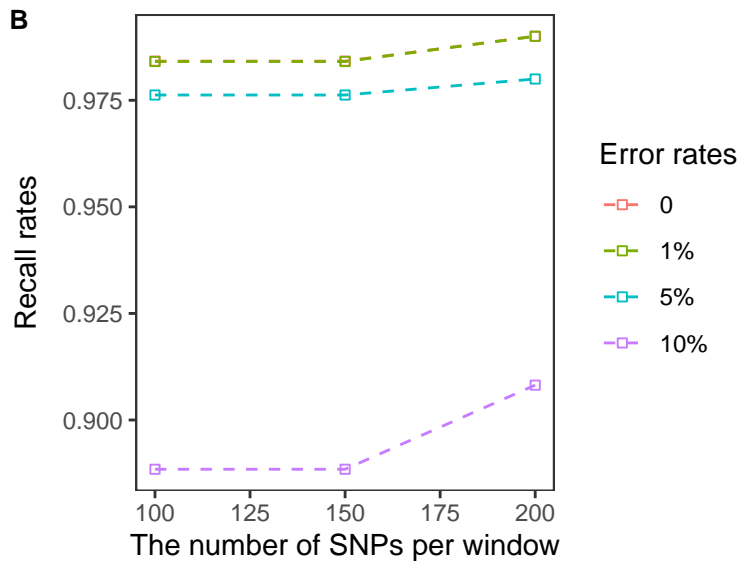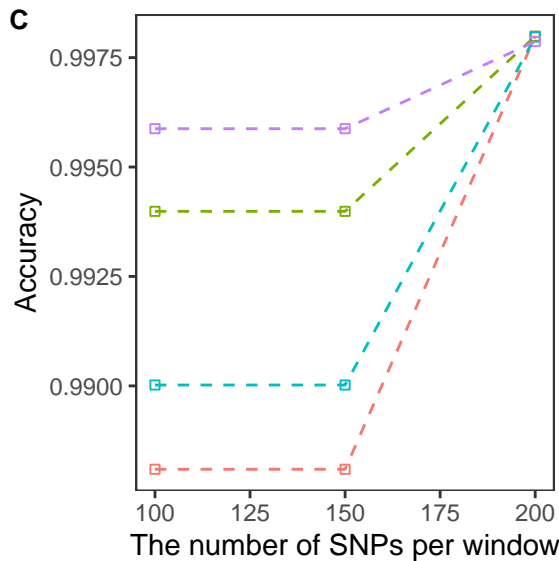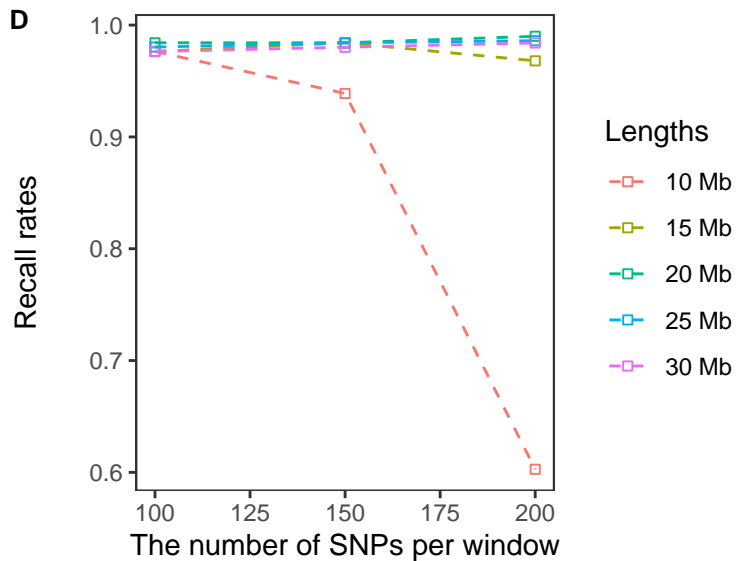

Supplement: qzaf055_Supplementary_Data [file qzaf055_supplementary_data.zip › Figure S5.pdf]

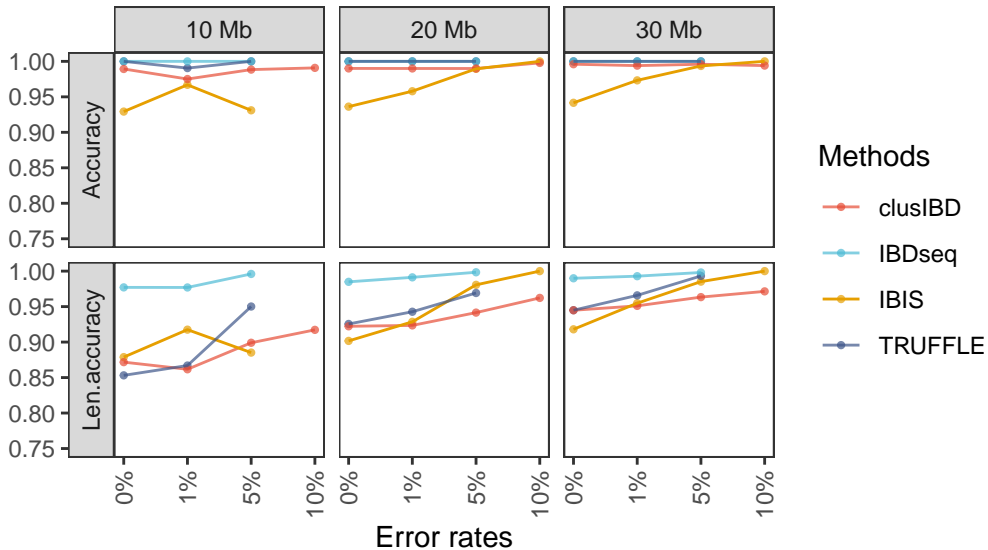

Supplement: qzaf055_Supplementary_Data [file qzaf055_supplementary_data.zip › Figure S6.pdf]

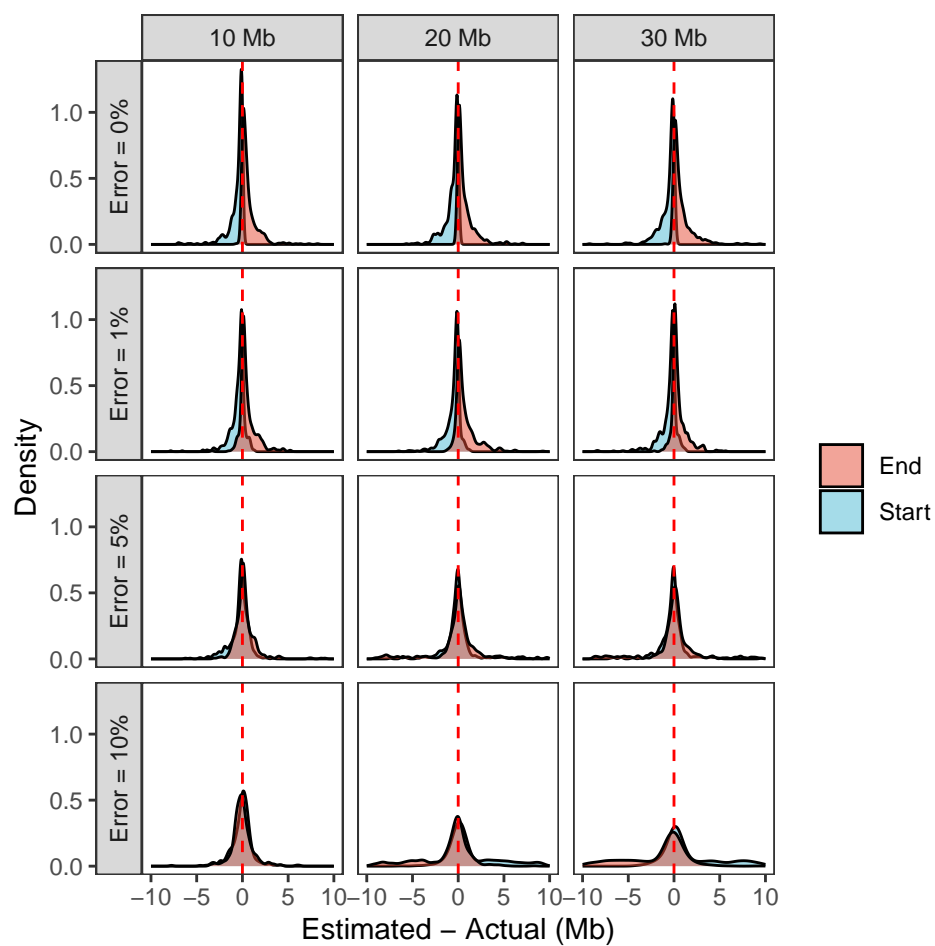

Supplement: qzaf055_Supplementary_Data [file qzaf055_supplementary_data.zip › Figure S7.pdf]

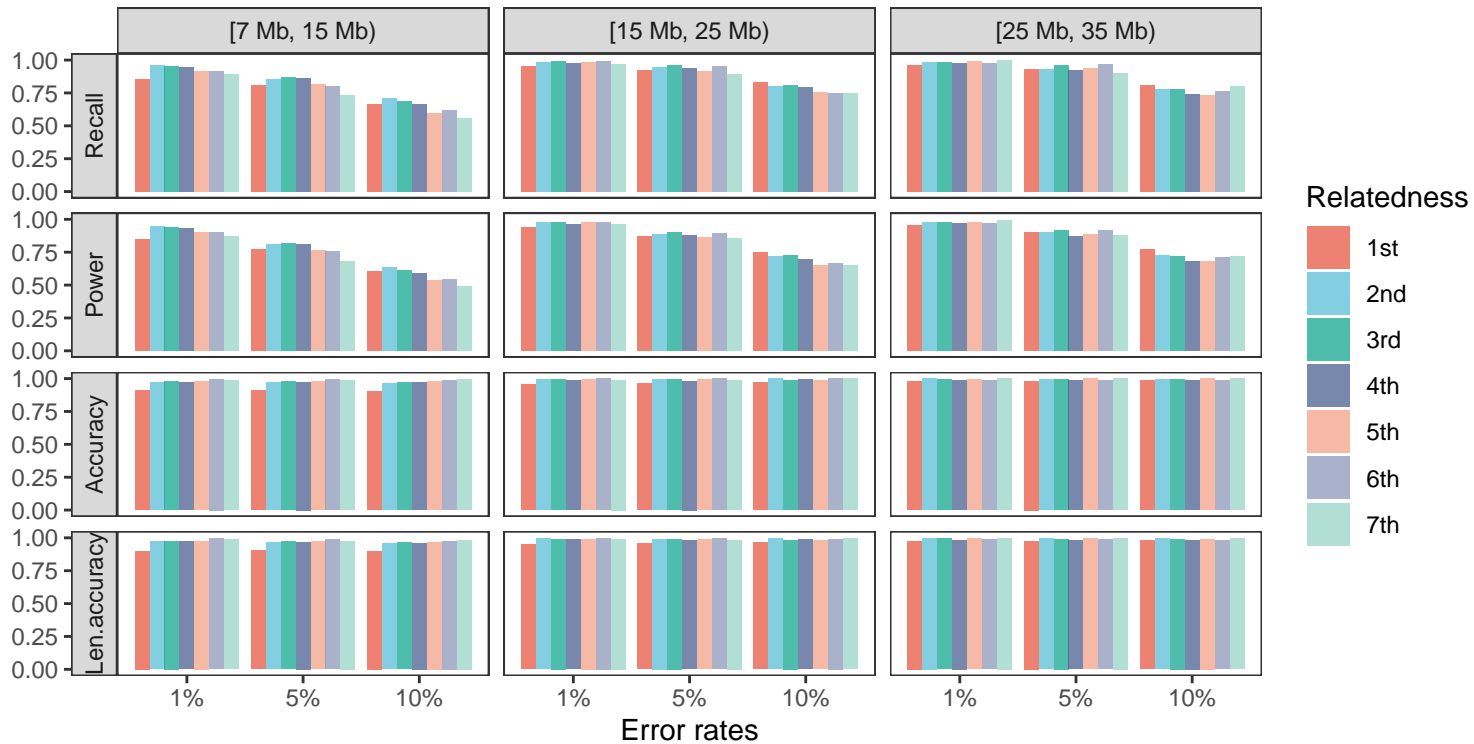

Supplement: qzaf055_Supplementary_Data [file qzaf055_supplementary_data.zip › Figure S8.pdf]

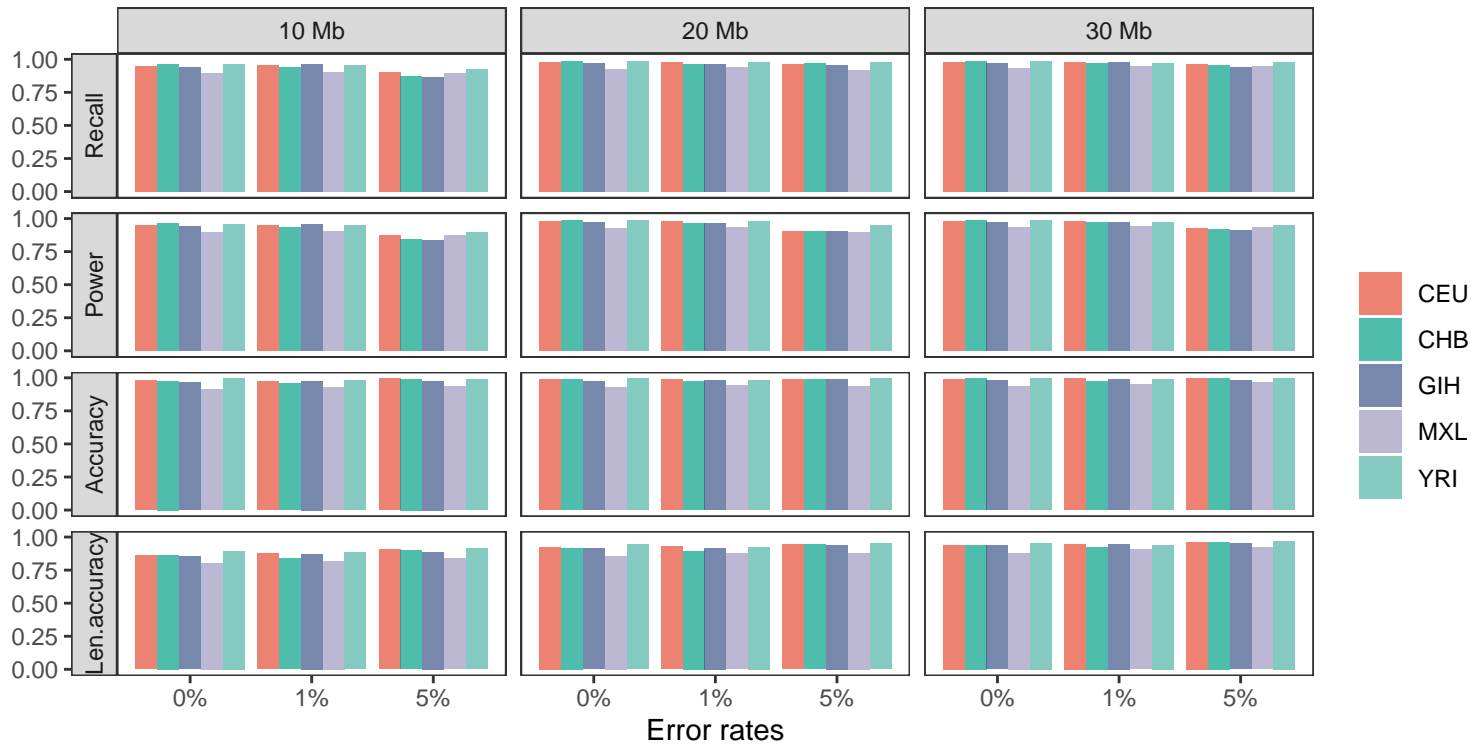

Supplement: qzaf055_Supplementary_Data [file qzaf055_supplementary_data.zip › Figure S9.pdf]
